# Mature Sunflower Inflorescences Face Geographical East to Maximize Absorbed Light Energy: Orientation of *Helianthus annuus* Heads Studied by Drone Photography

**DOI:** 10.3389/fpls.2022.842560

**Published:** 2022-03-17

**Authors:** Péter Takács, Zoltán Kovács, Dénes Száz, Ádám Egri, Balázs Bernáth, Judit Slíz-Balogh, Magdolna Nagy-Czirok, Zsigmond Lengyel, Gábor Horváth

**Affiliations:** ^1^Department of Biological Physics, Environmental Optics Laboratory, ELTE Eötvös Loránd University, Budapest, Hungary; ^2^Institute of Aquatic Ecology, Centre for Ecological Research, Budapest, Hungary; ^3^Estrato Research and Development Ltd., Budapest, Hungary; ^4^Department of Astronomy, ELTE Eötvös Loránd University, Budapest, Hungary; ^5^Szilády Áron Secondary School, Kiskunhalas, Hungary

**Keywords:** sunflower (*Helianthus annus*), inflorescence, east facing, sunrise, orientation, drone photography, environmental optics, photobiology

## Abstract

Mature sunflower (*Helianthus annuus*) inflorescences, which no longer follow the Sun, face the eastern celestial hemisphere. Whether they orient toward the azimuth of local sunrise or the geographical east? It was recently shown that they absorb maximum light energy if they face almost exactly the geographical east, and afternoons are usually cloudier than mornings. However, the exact average and standard deviation (SD) of the azimuth angle of the normal vector of mature sunflower inflorescences have never been measured on numerous individuals. It is imaginable that they prefer the direction of sunrise rather than that of the geographical east. To decide between these two photobiological possibilities, we photographed mature inflorescences of 14 sunflower plantations using a drone and determined the average and SD of the azimuth angle of the normal vector of 2,800 sunflower heads. We found that the average azimuth α_inflorescence_ = 89.5^°^ ± 42.8^°^ (measured clockwise from the geographical north) of inflorescences practically coincided with the geographical eastern direction (α_east_ = 90^°^) instead of the azimuth of local sunrise α_sunrise_ = 56.14^°^ – 57.55^°^. Although the SD of the orientation of individual inflorescences was large (± 42.8^°^), our finding experimentally corroborated the earlier theoretical prediction that the energetically ideal azimuth of sunflower inflorescences is east, if mornings are usually less cloudy than afternoons, which is typical for the domestication region of *H. annuus*. However, the average orientation of inflorescences of two plantations in hilly landscapes more or less differed from that of the majority of plantations in plane landscapes. The reason for this deviation may be that the illumination conditions in hilly sites more or less differed from those in plane landscapes. Furthermore, in a plantation, we observed a group of south-facing inflorescences that were in shadow for about 5 h both after sunrise and before sunset. This southern orientation can be explained by the southern maximum of total light energy absorbed by the partly shadowed inflorescences during the day, as computed by our software integrating both the diffuse skylight and the direct sunlight received by sunflower inflorescences.

## Introduction

The mature inflorescences of sunflowers (*Helianthus annuus* Linnaeus 1753) no longer follow the Sun (Shell and Lang, 1976; Lang and Begg, 1979), and their constant orientation influences flower temperature, number of pollinators, and plant fitness (Rey et al., 2008; Creux et al., 2021). It is a well-known phenomenon that they face approximately east (Darwin and Darwin, 1897; Vandenbrink et al., 2014; Kutschera and Briggs, 2016). In spite of this old knowledge, the exact average and standard deviation (SD) of the azimuth angle of the normal vector of mature sunflower inflorescences have never been measured on numerous plants. The obvious reason for this is that an *in situ* orientation measurement on several hundred/thousand sunflowers would be very difficult and time-consuming: In a sunflower plantation, the azimuth angle of many sunflower heads could be determined using a magnetic compass, for instance. Nowadays, this never-performed measurement can be replaced by the much easier drone photography.

Recently, Horváth et al. (2020) showed computationally that mature sunflower inflorescences absorb maximum light energy if they face almost exactly the geographical east, and afternoons are usually cloudier than mornings as is the case in eastern North America, which is the domestication region of *H. annuus* (Blackman et al., 2011). This result raises the following questions: Do mature inflorescences really orient precisely toward the geographical east? If yes, what is the SD of their average azimuth direction? Alternatively, it is photobiologically imaginable that they orient toward the azimuth of sunrise of the days when they cease to track the Sun. Notably, in the breeding season of sunflowers, the Sun rises/sets exactly at east/west only at the spring equinox (21 or 22 March). Before/after the spring equinox, the sunrise is at southeast/northeast and the sunset happens at southwest/northwest (Horváth et al., 2020). The main optical cue of the cessation of the partly light-governed mechanism of sunflower’s solar tracking (Shell and Lang, 1975; Atamian et al., 2016) could be the first direct sunlight originating from the local geographical azimuth direction of the sunrise. The advantage of such an orientation would be that inflorescences could receive direct sunlight as soon as possible after sunrise, and thus, the morning dew can quickly evaporate, which reduces the chance of fungal attack, as one of the hypotheses predicts (Lang and Begg, 1979).

To decide between the above-mentioned two photobiologically expectable orientations of sunflower heads, geographical east vs. azimuth of local sunrise, we photographed 2,800 mature inflorescences of 14 sunflower plantations using a drone and determined the average and SD of their azimuth angle. We found that the average azimuth of inflorescences is approximately east instead of the azimuth of sunrise. This finding experimentally corroborates the earlier theoretical prediction that the energetically ideal azimuth of sunflower inflorescences is east if mornings are generally less cloudy than afternoons, which is typical for the domestication region of *H. annuus* (Horváth et al., 2020).

## Materials and Methods

### Drone Photography of Sunflower Plantations

In southern and northern Hungary, we randomly selected 14 sunflower plantations for our drone photography campaign (Supplementary Table 1). The only criterion of selection was that the studied sunflowers should have mature inflorescences. We took 5 photographs of each plantation from heights ranging between 10 and 20 m, depending on the wind speed: at larger wind speeds, lower heights are optimal for the drone. For quantitative analysis, we chose the photographs of best quality.

**FIGURE 1:**
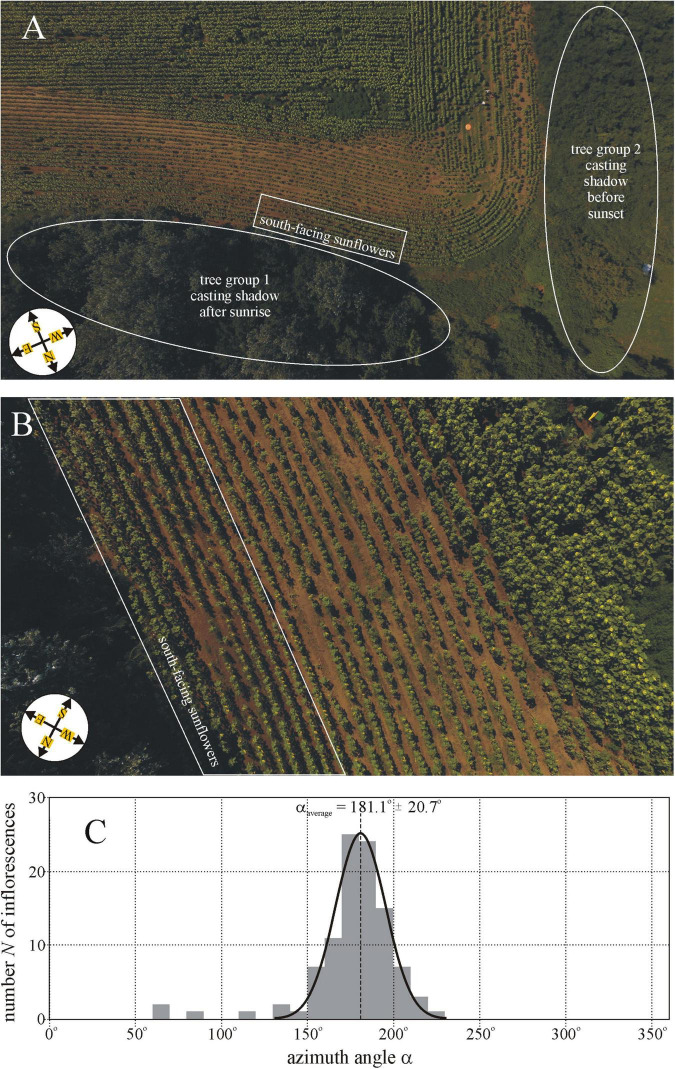
**(A)** Drone photograph of sunflower plantation 15 (Sződ, Supplementary Table 1) taken from a height of 80 m. The sunflower inflorescences in the white-perimeter rectangle face south rather than east as the other ones outside this rectangle. For about 5 h after sunrise, these sunflowers were in the shadow of tree group 1, while nearly 5 h prior to sunset they were shadowed by tree group 2. **(B)** Drone photograph of the same plantation taken from a height of 20 m. **(C)** Distribution of the azimuth angle α (measured clockwise from geographical north) of the normal vector of 100 mature sunflower inflorescences in the rectangle of Figure 1B. The Gaussian curve (characterized by peak azimuth α_average_ = 181.1^°^ and SD Δα_SD_ = ± 20.7^°^) is fitted to the *N*(α) graph. Drone photographs A and B are taken by Balázs Bernáth.

On July 7, 2020, we performed the first drone photography above a sunflower plantation near the Hungarian town Kiskunhalas (Supplementary Figure 1). The drone (DJI Phantom 3 Standard, field of view = 80^°^) captured photographs of mature sunflower inflorescences with its camera (DJI F300C). Using another drone (DJI Phantom 3 Advanced) with another camera (FC300S, field of view = 84^°^), in July and August 2021, we conducted a thorough drone photography campaign above 14 different Hungarian sunflower plantations (Figure 1 and Supplementary Figures 2–15). The optical axes of the camera of the drones were parallel to the local gravitational acceleration vector. Supplementary Table 1 summarizes the date, number, location, time, latitude, longitude, height, and camera yaw angle η (measured from the geographical north) of our drone campaigns. In all studied plantations, the hybrid type of the studied sunflowers (*H. annuus*) was the same: Cortewa (earlier named Pioneer) P64LE25.

### Evaluation of Drone Photographs of Mature Sunflower Heads

**FIGURE 2:**
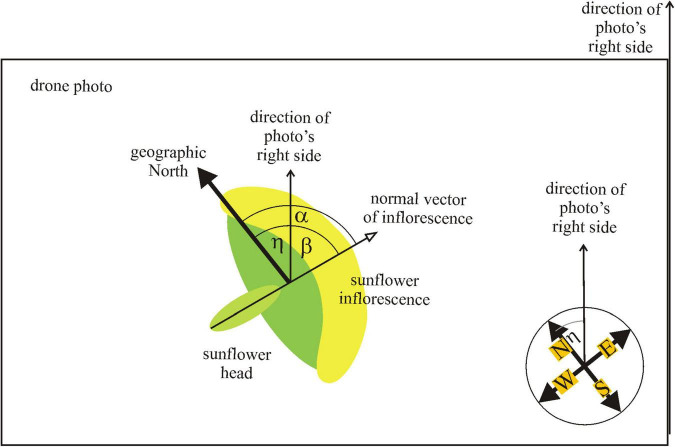
Definition of directions (normal vector of inflorescence, geographical north, and photograph’s right side) and angles (α, β, η) used during the evaluation of the drone photographs.

At the time of exposure of a photograph, the drone’s navigation system detected the yaw angle η between the drone’s inner reference direction ( = direction of the right side of the photo) and the geographical north (Supplementary Figures 1A,B). In the laboratory, we determined the direction of the normal vector of 200 randomly selected mature sunflower inflorescences in each of the drone photographs taken from 14 different sunflower plantations (Supplementary Table 1). We used a software developed by one of the authors (Ádám Egri), with which one can select a given inflorescence by clicking onto the center of the yellow arc of the flower’s petal series. Then a red circle appeared around the inflorescence with a red pointer (bar), the direction of which could be rotated arbitrarily. Using the computer’s mouse, the evaluator turned this pointer so that its direction became perpendicular to the inflorescence’s surface (Supplementary Figure 1C). Using this procedure, the evaluator estimated the angle β between the inflorescence’s normal vector and the direction of the photograph’s right side (Figure 2). The software registered the value of β and waited for the manual-visual evaluation of the next selected inflorescence. Finally, the azimuth angle α = β + η of the inflorescence’s normal vector measured from the north was obtained (Figure 2).

### Computation of the Energy-Maximizing Azimuth of Sunflower Inflorescences

To explain the phenomenon of the southern orientation of a group of sunflowers shadowed for 5 h both after sunrise and before sunset, we used the computer program developed by Horváth et al. (2020). This software computes the total light energy *e* per unit area absorbed by a sunflower inflorescence between anthesis (1 July) and senescence (7 September) as a function of the azimuth angle α of its normal vector for the typical cloud conditions of a given region, in our present case for Hungary, where mornings are less cloudy than afternoons. It uses local astronomical data of the celestial motion of the Sun and regional meteorological data of diurnal cloudiness between anthesis and senescence, furthermore the time-dependent elevation angle of mature sunflower heads and the absorption spectra of inflorescences. More details about this computational method can be found in the study by Horváth et al. (2020).

## Results

### Typical Distribution of the Azimuth Angle of Sunflower Inflorescences

Figure 3 shows the distribution *N*(α) of the azimuth angle α (measured clockwise from geographical north) of the normal vector of 2,800 ( = 14 × 200) randomly selected mature sunflower inflorescences at locations 1–14 (Supplementary Table 1) determined in the drone photographs (Supplementary Figures 2–15), where *N* is the number of inflorescences. The average ± SD of the Gaussian curve fitted to the *N*(α) graph are α_average_ = 89.5^°^ ± 42.8^°^. α_average_ practically coincided with the geographical east α_east_ = 90^°^.

**FIGURE 3:**
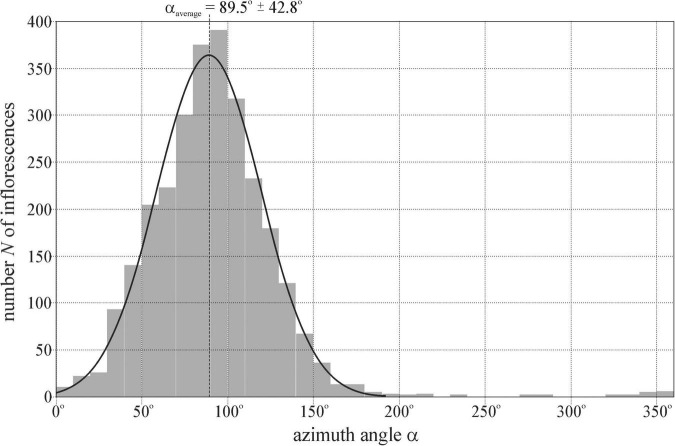
Distribution of the azimuth angle α (measured clockwise from geographical north) of the normal vector of 2800 ( = 14 × 200) randomly selected mature sunflower inflorescences studied at locations 1–14 (Supplementary Table 1) determined in the drone photographs of Supplementary Figures 2–15. The Gaussian curve (characterized by peak azimuth α_average_ = 89.5^°^ and SD Δα_SD_ = ± 42.8^°^) is fitted to the *N*(α) graph.

Table 1 contains the average azimuth α_average_ ± SD Δα_SD_ (measured clockwise from geographical north) of the normal vector of the randomly selected mature sunflower inflorescences at locations 1–14 (Supplementary Figures 16–29 and Supplementary Table 1) determined in the drone photographs of Supplementary Figures 2–15, and the values of the azimuth α_sunrise_ of local sunrise. According to Table 1, at locations 1–5 and 7–13, the average azimuth angle α_average_ of sunflower inflorescences changed between 77.6 and 98.3^°^, and the minimal and maximal SD were Δα_SD_(min) = 24.9^°^ and Δα_SD_(max) = 56.6^°^. The minimum and maximum values of the difference Δ_east–average_ = α_east_ −α_average_ between α_average_ and the azimuth α_east_ = 90^°^ of geographical east were Δ_east–average_(min) = 1.1^°^ and Δ_east–average_(max) = 12.4^°^, respectively. Since in these cases Δ_east–average_ was much smaller than Δα_SD_, we conclude that the average azimuth angle α_average_ of the normal vector of these sunflower inflorescences did not deviate significantly from the geographical eastern direction α_east_.

**TABLE 1:** Average azimuth α_average_ ± standard deviation (SD) Δα_SD_ (measured clockwise from geographical north), Δ_east–average_ = α_east_ ( = 90^°^) −α_average_, and Δ_average–sunrise_ = α_average_ −α_sunrise_ for the normal vector of randomly selected 200 mature sunflower inflorescences at locations 1–14 (Supplementary Figures 16–29 and Supplementary Table 1) determined in the drone photographs of Supplementary Figures 2–15.

Location	α_average_	Δα_SD_	Δ_east–average_ = α_east_ −α_average_	α_sunrise_	Δ_average–sunrise_ = α_average_ −α_sunrise_
(1) Kiskunhalas	87.9^°^	± 24.9^°^	2.1^°^	56.14^°^	31.76^°^
(2) Sződ 1	77.6^°^	± 46.1^°^	12.4^°^	56.66^°^	20.94^°^
(3) Sződ 2	94.6^°^	± 43.3^°^	4.6^°^	56.66^°^	37.94^°^
(4) Sződ 3	94.2^°^	± 50.5^°^	4.2^°^	56.66^°^	37.54^°^
(5) Sződ 4	86.4^°^	± 56.6^°^	3.6^°^	56.66^°^	29.74^°^
(6) Vácduka 1	66.4^°^	± 51.5^°^	23.6^°^	56.65^°^	9.75^°^
(7) Vácduka 2	79.9^°^	± 54.2^°^	10.1^°^	56.65^°^	23.25^°^
(8) Környe 1	79.6^°^	± 36.9^°^	10.4^°^	57.52^°^	22.08^°^
(9) Környe 2	98.3^°^	± 34.0^°^	8.3^°^	57.52^°^	40.78^°^
(10) Környe 3	91.1^°^	± 38.7^°^	1.1^°^	57.52^°^	33.58^°^
(11) Környe 4	80.1^°^	± 45.6^°^	9.9^°^	57.52^°^	22.58^°^
(12) Környe 5	88.1^°^	± 28.4^°^	1.9^°^	57.52^°^	30.58^°^
(13) Környe 6	97.3^°^	± 25.1^°^	7.3^°^	57.52^°^	39.78^°^
(14) Környe 7	114.4^°^	± 39.5^°^	−24.4^°^	57.55^°^	56.85^°^
averaged for all 14 locations	89.5^°^	± 42.8^°^	0.5^°^	−	−
(15) Sződ 5 (facing south)	181.1^°^	± 20.7^°^	−91.1^°^	62.97^°^	118.13^°^

*α_sunrise_ is the azimuth of local sunrise at locations 1–14 during our drone photography campaigns. The last row contains the data of south-facing inflorescences at location 15.*

In the studied sunflower plantations, the sun rose at azimuth angles 56.14^°^ ≤ α_sunrise_ ≤ 57.55^°^, during our drone photography campaigns. According to Table 1, at locations 1–5 and 7–13 the minimum and maximum values of the difference Δ_average–sunrise_ = α_average_ −α_sunrise_ between α_average_ and the azimuth α_sunrise_ of local sunrise were Δ_average–sunrise_ (min) = 20.94^°^ and Δ_average–sunrise_ (max) = 40.78^°^, respectively. Since in these locations Δ_average–sunrise_ was always much larger than Δ_east–average_, we conclude that the average azimuth angle α_average_ of the normal vector of these sunflower inflorescences deviated significantly from the azimuth direction α_sunrise_ of local sunrise.

### Atypical Orientation of Sunflower Heads

However, locations 6 and 14 are exceptions from these trends: At location 6, α_average_ = 66.4^°^ ± 51.5^°^ differed only by Δ_average–sunrise_ = 9.75^°^ from α_sunrise_ = 56.65^°^ and differed by Δ_east–average_ = 23.6^°^ from α_east_ = 90^°^ (Table 1). Here, the SD Δα_SD_ = 51.5^°^ was much larger than Δ_east–average_ = 23.6^°^, and Δ_average–sunrise_ = 9.75^°^ was relatively small. From these, we conclude that although the average azimuth of these sunflower inflorescences did not differ significantly from the eastern azimuth α_east_ = 90^°^, it was much closer to the north-eastern azimuth of local sunrise.

At location 14, α_average_ = 114.4^°^ ± 39.5^°^ differed by Δ_average–sunrise_ = 56.85^°^ from α_sunrise_ = 57.55^°^ and differed by Δ_east–average_ = −24.4^°^ from α_east_ = 90^°^ (Table 1). Here, since the SD Δα_SD_ = 39.5^°^ was much larger than | Δ_east–average_| = 24.4^°^ and Δ_average–sunrise_ = 56.85^°^ was relatively large, we conclude that the average azimuth of these sunflower inflorescences did not differ significantly from the eastern azimuth α_east_ = 90^°^ and differed significantly from the azimuth of local sunrise. Nevertheless, the deviation Δ_east–average_ = −24.4^°^ of α_average_ from α_east_ toward south-east was at least two times larger than at locations 1–5 and 7–13.

The reason for these two last deviations at locations 6 and 14 from the general trends at locations 1–5 and 7–13 positioned on horizontal plane landscapes may be that the sunflower plantations 5 and 14 were in hilly landscapes where the illumination received by sunflower inflorescences more or less differed from that at flat sites.

### Tree-Shadowed Sunflower Inflorescences Facing South

In sunflower plantation 15 (Sződ 5, Supplementary Table 1), we observed that a group of mature inflorescences faced south rather than east as the majority of sunflowers. For about 5 h after sunrise, this inflorescence group was in the shadow of tree group 1 shown in Figure 1A, while for nearly 5 h prior to sunset it was shadowed by tree group 2 (Figure 1A). Figure 1C shows the distribution of the azimuth angle α (measured clockwise from geographical north) of the normal vector of 100 mature sunflower inflorescences in the white-perimeter rectangle of Figure 1B. The majority of these inflorescences faced practically south (α_average_ = 181.1^°^ ≈α_South_ = 180^°^) with SD Δα = ± 20.7^°^, and the minimum and maximum of their azimuth were α_min_ = 63^°^ and α_max_ = 219^°^, respectively (Figure 1C).

### Radiational Reason for the South-Facing of Sunflowers

**FIGURE 4:**
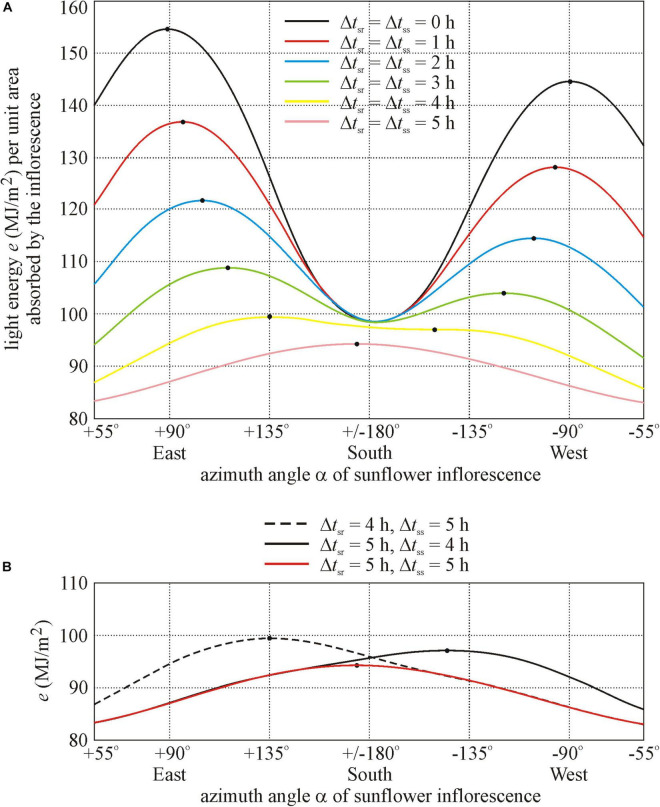
Total light energy *e* (MJ/m^2^) per unit area absorbed by a sunflower inflorescence between anthesis (1 July) and senescence (7 September) as a function of the azimuth angle α (measured clockwise from geographical north) of its normal vector computed for Hungary, if the inflorescence receives direct sunlight only in the time interval *t*_sr_ + Δ*t*_sr_ ≤ *t* ≤ *t*_ss_ −Δ*t*_ss_, where *t*_sr_ and *t*_ss_ are the times of sunrise and sunset and Δ*t*_sr_ and Δ*t*_ss_ are the shadowed periods after sunrise and before sunset, respectively. The inflorescence is illuminated continuously by diffuse skylight between sunrise and sunset. The primary and secondary maxima of curves *e*(α) are marked by dots. **(A)** Δ*t*_sr_ = Δ*t*_ss_ = 0, 1, 2, 3, 4, and 5 h. **(B)** Δ*t*_sr_ = 4 h and Δ*t*_ss_ = 5 h, Δ*t*_sr_ = 5 h and Δ*t*_ss_ = 4 h, Δ*t*_sr_ = Δ*t*_ss_ = 5 h.

To find a possible reason for the southern orientation of these sunflowers, we computed the total light energy *e* per unit area absorbed by a sunflower inflorescence between anthesis (1 July) and senescence (7 September) as a function of the azimuth angle α of its normal vector. We used the computer program developed by Horváth et al. (2020) for the typical cloud conditions in Hungary, where mornings are usually less cloudy than afternoons. Since the south-facing sunflowers in Figure 1 were in the shadow of trees both after sunrise and before sunset for approximately 5 h, in our computations we assumed that (i) the model inflorescence was illuminated continuously by diffuse skylight between sunrise and sunset (ii) and it received direct sunlight only in the time interval *t*_sr_ + Δ*t*_sr_ ≤ *t* ≤ *t*_ss_ −Δ*t*_ss_, where *t*_sr_ and *t*_ss_ are the times of sunrise and sunset and Δ*t*_sr_ and Δ*t*_ss_ are the periods after sunrise and before sunset, respectively. The computational results for different illumination conditions are shown in Figure 4:

• Where a sunflower inflorescence is not shadowed between sunrise and sunset (Δ*t*_sr_ = Δ*t*_ss_ = 0 h), it receives maximal light energy *e* if its normal vector points toward the eastern azimuth angle α_east_ = 90^°^ (see the black curve *e*(α) in Figure 4A).
• Where an inflorescence is temporally symmetrically shadowed for a period Δ*t*_sr_ = Δ*t*_ss_ = 1 or 2 h both after sunrise and before sunset, it receives maximal energy if its normal vector points toward the south-eastern azimuth α = 95^°^ or 104^°^, respectively (red and blue curves in Figure 4A).
• Where an inflorescence is shadowed for a period Δ*t*_sr_ = Δ*t*_ss_ = 3 h both after sunrise and before sunset, it receives maximal energy if its normal vector points toward the south-eastern azimuth α = 116^°^ (green curve in Figure 4A).
• Where an inflorescence is shadowed for a period Δ*t*_sr_ = Δ*t*_ss_ = 4 h both after sunrise and before sunset, it receives maximal energy if its normal vector points toward the south-eastern azimuth α = 135^°^ (yellow curve in Figure 4A).
• Where an inflorescence is shadowed for a period Δ*t*_sr_ = Δ*t*_ss_ = 5 h both after sunrise and before sunset, it receives maximal energy if its normal vector points toward the nearly southern azimuth α = 174^°^ (violet curve in Figure 4A). This is the situation that can explain the southern orientation of sunflower inflorescences in Figure 1. According to Figure 4A, in this case (violet curve in Figure 4A), the energy absorbed by the inflorescence is much smaller than that in the other, less shadowed situations (yellow, green, blue, red, and black curves in Figure 4A). Remarkably, these south-facing sunflowers developed their inflorescences 2 weeks later than the non-shadowed ones, and the height of the formers was about half of that of the latter. One of the reasons for this may be the much less direct sunlight due to the 5 h morning and 5 h crepuscular shading by trees.
• According to Figure 4B, the energy-maximizing azimuth angle of a temporally asymmetrically shadowed inflorescence is α = 135^°^ (black dashed curve) when Δ*t*_sr_ = 4 h and Δ*t*_ss_ = 5 h, while it is α = −144^°^ (black continuous curve) when Δ*t*_sr_ = 5 h and Δ*t*_ss_ = 4 h.

## Discussion

For the 2,800 randomly selected sunflowers of hybrid type Cortewa P64LE25 in sunflower plantations 1–14, we found that the average azimuth of inflorescences coincided with the geographical eastern direction and differed significantly from the azimuth of local sunrise. This experimentally corroborates the earlier theoretical prediction that the energetically ideal azimuth of sunflower inflorescences is east if mornings are usually less cloudy than afternoons, which is typical for the domestication region of *H. annuus*.

However, we found three exceptions to this rule. In two plantations positioned in hilly landscapes, the average orientation of inflorescences more or less differed from that (east) of the majority of plantations in plane landscapes. We assumed that the reason for this deviation could be the deviating illumination conditions of plantations positioned on the tilted ground of hilly sites compared with those of plantations in plane landscapes. In a third plantation, we could quantitatively explain why a group of inflorescences faced south: They were shadowed for 5 h both after sunrise and before sunset, thus the total light energy absorbed by them during the day was maximal for south facing rather than east facing. This case study also demonstrates that the local illumination conditions strongly determine the energy-maximizing ideal azimuth orientation of mature sunflower inflorescences.

We determined the azimuth angle α_sunrise_ of sunrise for the days on which the drone photographs were taken at the studied 15 locations (Table 1). However, the anthesis, when the azimuth angle α of the normal vector of sunflower inflorescences has come to stay, happened about 1 week earlier than our drone photography. Seven days before anthesis, α_sunrise_ was approximately 2.5^°^ smaller, which does not influence our conclusions.

**FIGURE 5:**
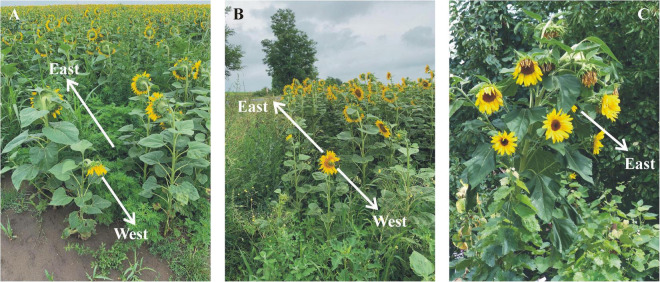
**(A,B)** A west-facing low sunflower at the western edge of sunflower plantations 8 **(A)** and 9 **(B)** near Környe (Supplementary Table 1). **(C)** A wild-type *Helianthus annuus* sunflower with one primary and 8 secondary inflorescences. Photographs A and B are taken by Péter Takács, while C is taken by Gábor Horváth.

For the investigated sunflowers, we found that the maximal scatter (not the SD) of the azimuth direction of inflorescences approximated ± 90^°^. However, at the western edge of two plantations, we found a mature, pauperitic, low sunflower, the inflorescence of which faced the geographical west (Figures 5A,B). Both low sunflowers were in the shadow of the neighboring tall sunflowers until early afternoon. Their western orientation (deviating extremely from the east) is a further example of the strong influence of shading.

Nevertheless, such a great disorientation is very rare. All these mean that in a sunflower plantation the inflorescence of many plants can face nearly or precisely the geographical north or south rather than the ideal east. There can be at least four biological/environmental reasons for such large disorientations: an observationally and computationally proven environmental reason is that certain sunflowers are shadowed by the neighboring vegetation for some periods during the day, as demonstrated by the south-facing sunflowers in Figures 1, 4. A second reason can be that a later developing and flowering smaller sunflower is in the shadow of its larger, more developed neighbors for some periods of the day (Figures 5A,B). The orientational consequence of this shadowing is similar to that articulated above. The fixed direction of a shadowed inflorescence depends on the local diurnal illumination conditions determined by the sunlit and shadowed periods throughout the day. A third reason may be a dysfunction of the gene expression of the genetically coded ideal eastward orientation. Such a dysfunction could also be induced by certain environmental factors, such as the local mineral composition as well as nutrient- and moisture content of the soil, for example. A fourth reason could be a dysfunction of the circadian clock that plays a crucial role in the regulation of the tracking motion and the final eastern orientation of the capitulum (Atamian et al., 2016). Such a dysfunction could be induced by the erroneous expression and/or mutation of the circadian clock genes, for instance. Although the experimental study of these possibilities is out of the scope of this study, in the future it would be worth investigating them.

We emphasize that our results are valid only for sunflowers having only one inflorescence. It is well-known that wild-type sunflowers develop one primary and several secondary inflorescences (Figure 5C). Since the latter grow usually from the radix of the leaf stalk, their orientation is predominantly determined by the direction of the radix, which can be arbitrary.

## Data Availability Statement

The original contributions presented in the study are included in the article/Supplementary Material, further inquiries can be directed to the corresponding author.

## Author Contributions

PT, JS-B, and GH substantial contributions to conception and design. PT, ÁE, and JS-B software development. PT, DS, BB, JS-B, MN-C, ZL, and GH performing experiments and measurements. PT, ZK, ÁE, BB, ZL, and GH data visualization. PT, ZK, DS, and GH data analysis and interpretation. PT, DS, and GH drafting the article and revising it critically. All authors contributed to the article and approved the submitted version.

## Conflict of Interest

BB was employed by the company Estrato Research and Development Ltd. The remaining authors declare that the research was conducted in the absence of any commercial or financial relationships that could be construed as a potential conflict of interest.

## Publisher’s Note

All claims expressed in this article are solely those of the authors and do not necessarily represent those of their affiliated organizations, or those of the publisher, the editors and the reviewers. Any product that may be evaluated in this article, or claim that may be made by its manufacturer, is not guaranteed or endorsed by the publisher.
