# Supplementary material for: Mature Sunflower Inflorescences Face Geographical East to Maximize Absorbed Light Energy: Orientation of Helianthus annuus Heads Studied by Drone Photography
Source: Front Plant Sci. 2022 Mar 17;13:842560. doi: 10.3389/fpls.2022.842560 (PMC8969559; doi:10.3389/fpls.2022.842560)
Supplement: Supplementary file 1 [file Data_Sheet_1.doc]

**Supplementary Material**

for

**Mature sunflower inflorescences face geographical east to maximize absorbed light energy: orientation of *Helianthus annuus* heads studied by drone photography**

Péter Takács, Zoltán Kovács, Dénes Száz, Ádám Egri, Balázs Bernáth, Judit Slíz-Balogh,

Magdolna Nagy-Czirok, Zsigmond Lengyel and Gábor Horváth^1,*^

1: Environmental Optics Laboratory, Department of Biological Physics,

ELTE Eötvös Loránd University, H-1117 Budapest, Pázmány sétány 1, Hungary

^*^Corresponding author: gh@arago.elte.hu

This file contains the following: - Supplementary Table S1,

- Supplementary Figures S1-S29

**Supplementary Table S1**: Date, number (No.), location, time (Greenwich Mean Time + 2 hours), northern latitude, eastern longitude, height *h* (m) and camera’s yaw angle η (measured from the geographical north) of the drone photographs taken about 15 different Hungarian sunflower plantations. The left-right margins of a drone photo is turned by η clockwise from the geographical north (Figure 1D). GMT: Greenwich Mean Time. Height *h* is measured from the average plane of sunflower heads.

| **date** | **No.** | **location**  **(Hungary)** | **filename** | **time**  **(GMT**  **+ 2h)** | **northern**  **latitude** | **eastern**  **longitude** | **height**  ***h* (m)** | **camera**  **yaw η** |
| --- | --- | --- | --- | --- | --- | --- | --- | --- |
| 2020  7 July | 1. | Kiskunhalas | Kiskunhalas_DJI_3.jpg | 19:33 | 46^o^ 27' 9.10" | 19^o^ 31' 3.92" | 20 | +127.5^o^ |
| 2021 |  |  |  |  |  |  |  |  |
| 14 July | 2. | Sződ 1 | Szod_1_1_20_DJI_0033.jpg | 12:39:19 | 47^o^ 43' 32.02" | 19^o^ 12' 0.73" | 20 | -2.2^o^ |
| 14 July | 3. | Sződ 2 | Szod_1_2_20_DJI_0075.jpg | 12:42:44 | 47^o^ 43' 30.47" | 19^o^ 12' 3.11" | 20 | -2.1^o^ |
| 14 July | 4. | Sződ 3 | Szod_2_1_20_DJI_0007.jpg | 11:33:40 | 47^o^ 43' 35.77" | 19^o^ 11' 57.69" | 20 | +0^o^ |
| 14 July | 5. | Sződ 4 | Szod_2_2_20_DJI_0032.jpg | 12:18:02 | 47^o^ 43' 35.19" | 19^o^ 12' 0.75" | 20 | +7.7^o^ |
| 14 July | 6. | Vácduka 1 | Vacduka_1_10_DJI_0121.jpg | 13:24:08 | 47^o^ 44' 3.82" | 19^o^ 13' 12.03" | 10 | +1.8^o^ |
| 14 July | 7. | Vácduka 2 | Vacduka_2_10_DJI_0033.jpg | 13:46:48 | 47^o^ 44' 5.04" | 19^o^ 13' 10.42" | 10 | +1.8^o^ |
| 17 July | 8. | Környe 1 | Kornye_1_15_DJI_0011.jpg | 11:21:30 | 47^o^ 37' 6.35" | 18^o^ 19' 2.24" | 15 | -2.5^o^ |
| 17 July | 9. | Környe 2 | Kornye_2_20_DJI_0032.jpg | 11:23:28 | 47^o^ 37' 4.91" | 18^o^ 19' 6.60" | 20 | +52.3^o^ |
| 17 July | 10. | Környe 3 | Kornye_3_15_DJI_0015.jpg | 11:55:02 | 47^o^ 37' 3.35" | 18^o^ 18' 56.30" | 15 | -140.1^o^ |
| 17 July | 11. | Környe 4 | Kornye_4_20_DJI_0002.jpg | 12:20:54 | 47^o^ 36' 55.38" | 18^o^ 19' 11.32" | 20 | +31.4^o^ |
| 17 July | 12. | Környe 5 | Kornye_5_15_DJI_0046.jpg | 12:24:16 | 47^o^ 36' 51.15" | 18^o^ 19' 9.26" | 15 | +72.6^o^ |
| 17 July | 13. | Környe 6 | Kornye_6_15_DJI_0078.jpg | 12:27:12 | 47^o^ 36' 53.53" | 18^o^ 19' 2.87" | 15 | -51.6^o^ |
| 17 July | 14. | Környe 7 | Kornye_7_15_DJI_0012.jpg | 13:10:02 | 47^o^ 34' 46.17" | 18^o^ 19' 22.16" | 15 | +136.4^o^ |
| 2 August | 15. | Sződ 5 | Szod_3_20_DJI_0043.jpg | 09:52:47 | 47^o^ 43' 32.40" | 19^o^ 12' 0.64" | 20 | +151.4^o^ |


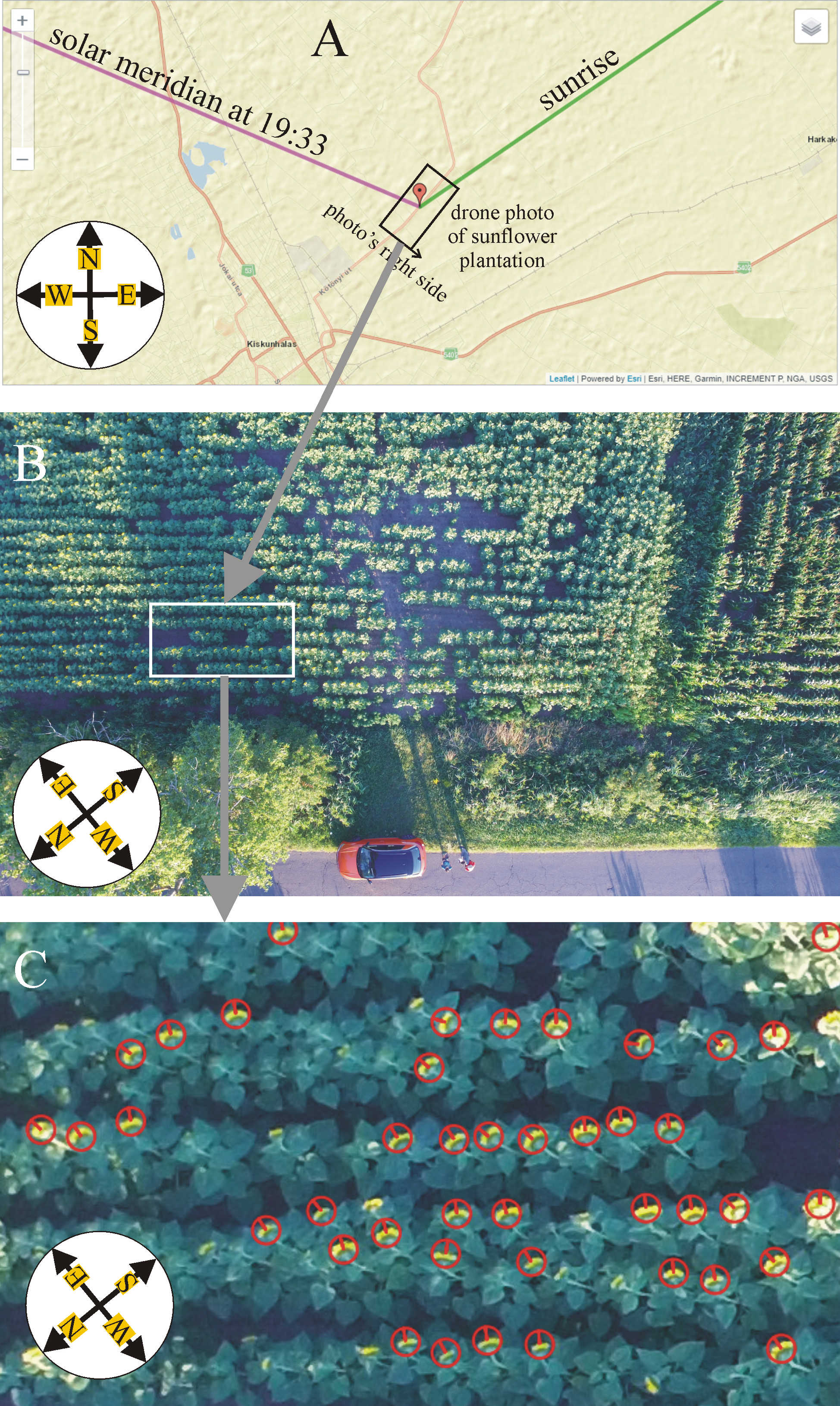


**Supplementary Figure S1:** (A) Location of the studied sunflower plantation near Kiskunhalas (south Hungary) depicted in a Google map together with the directions of the solar meridian at 19:33 (= local summer time = GMT + 2 hours) on 7 July 2020 when the drone photography was performed (violet line), and of the sunrise (green line) on the same day. (B) Drone photo of the sunflower plantation near an asphalt road. η is the drone’s yaw angle from the geographical north and is equal to the angle between north and the direction of the photo’s right side. (C) Some evaluated sunflower heads, where the red pointers (bars) in the red circles show the visually estimated normal vectors of inflorescences. Map A originates from https://gml.noaa.gov/grad/solcalc/, while the drone photos B and C were taken by Zsigmond Lengyel.


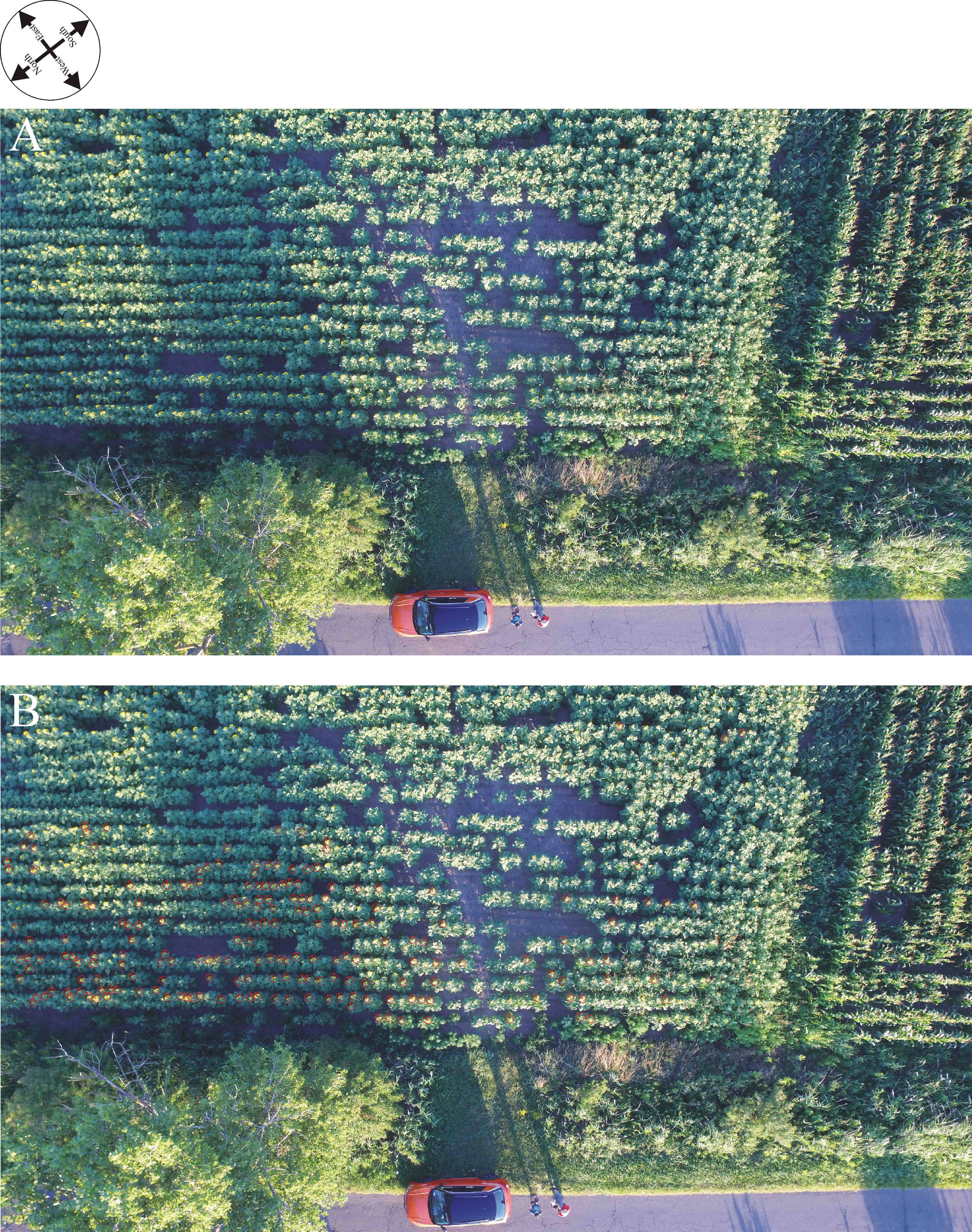


**Supplementary Figure S2**: Drone photographs of the sunflower plantation at location 1 (Kiskunhalas, Supplementary Table S1) taken by Zsigmond Lengyel. (A) Original drone photo. (B) Drone photo in which the 200 evaluated sunflower heads are marked by red circles and the normal vectors of their inflorescences are depicted by a red bar.


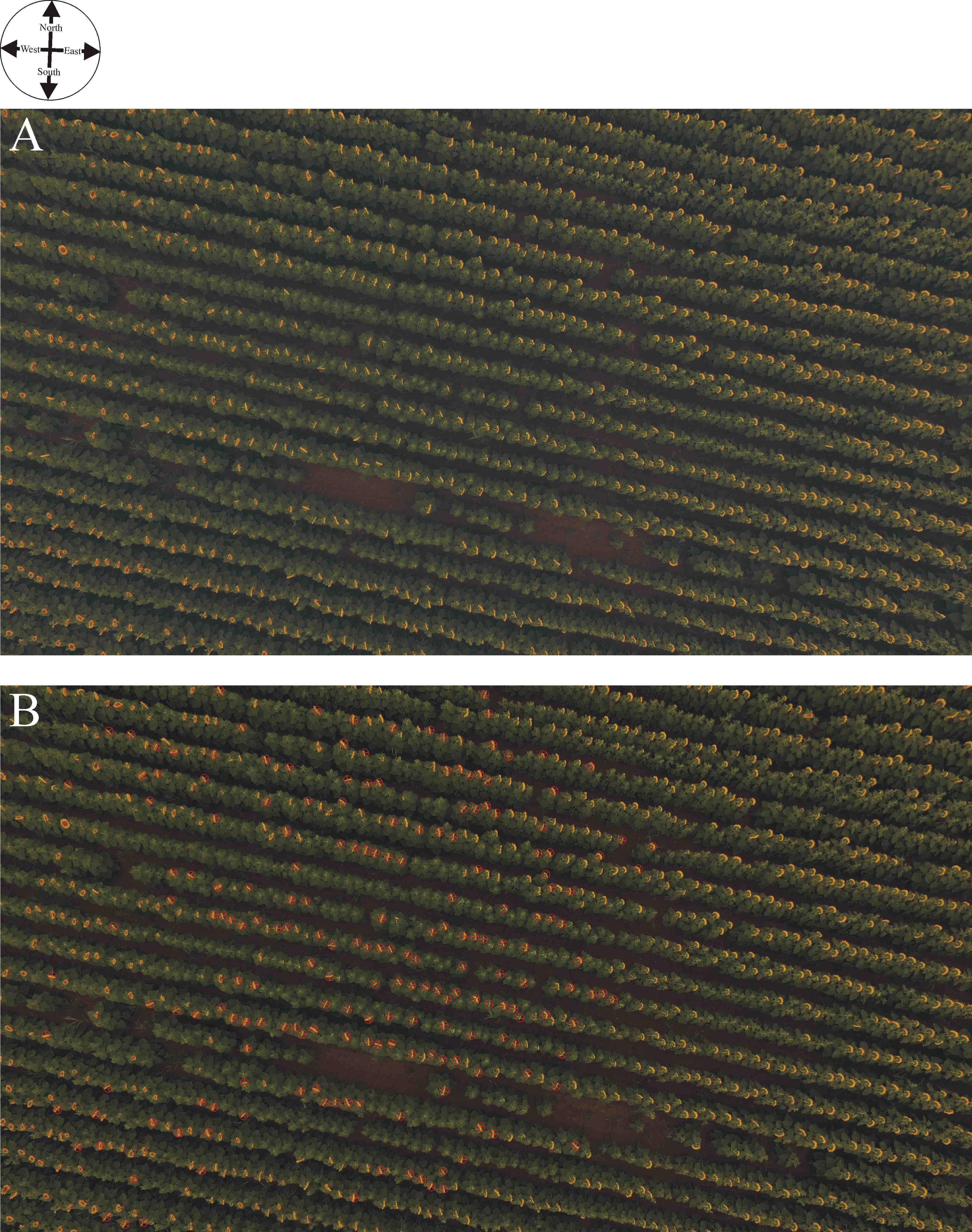


**Supplementary Figure S3**: Drone photographs of the sunflower plantation at location 2 (Sződ 1, Supplementary Table S1) taken by Balázs Bernáth. (A) Original drone photo. (B) Drone photo in which the 200 evaluated sunflower heads are marked by red circles and the normal vectors of their inflorescences are depicted by a red bar.


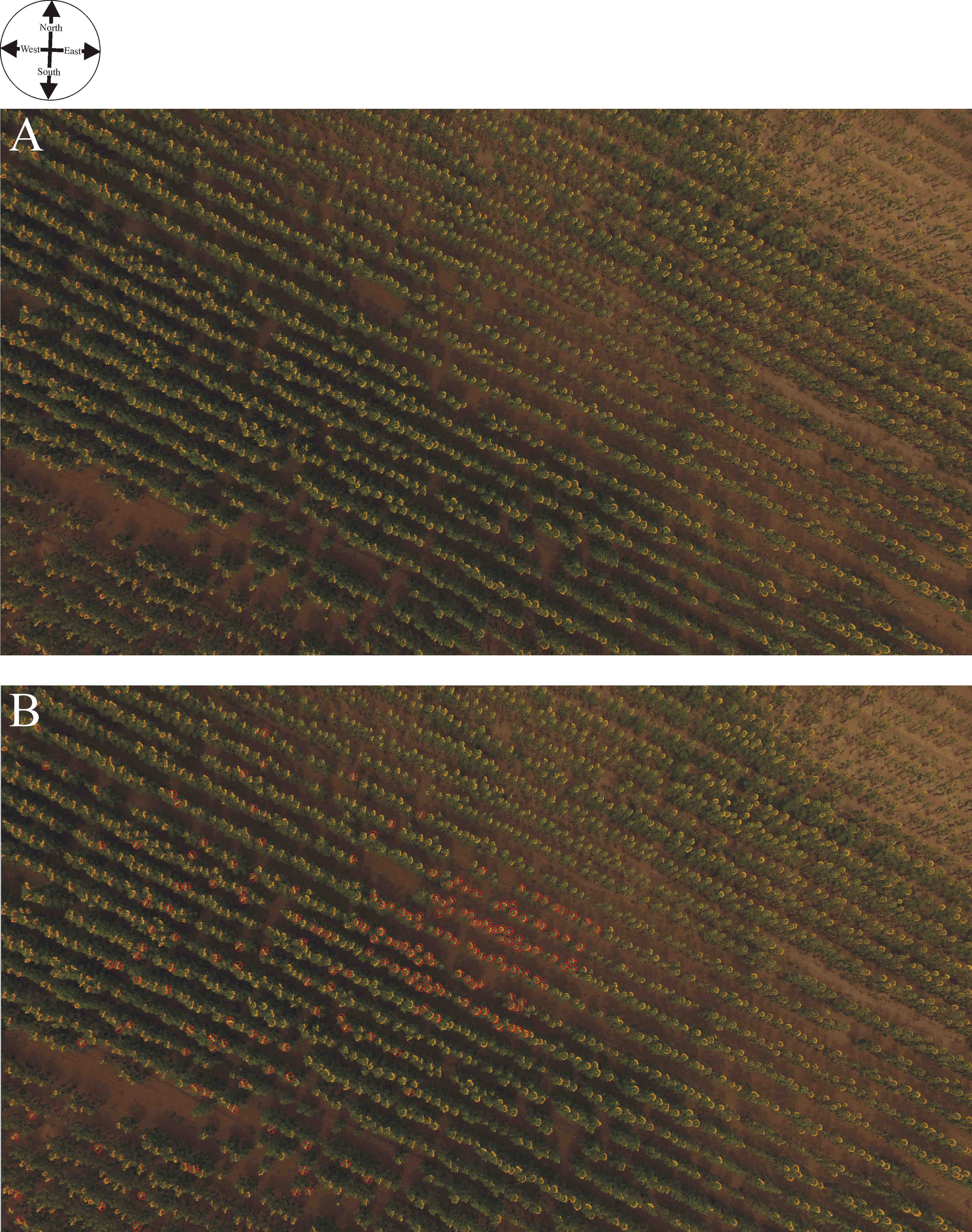


**Supplementary Figure S4**: Drone photographs of the sunflower plantation at location 3 (Sződ 2, Supplementary Table S1) taken by Balázs Bernáth. (A) Original drone photo. (B) Drone photo in which the 200 evaluated sunflower heads are marked by red circles and the normal vectors of their inflorescences are depicted by a red bar.


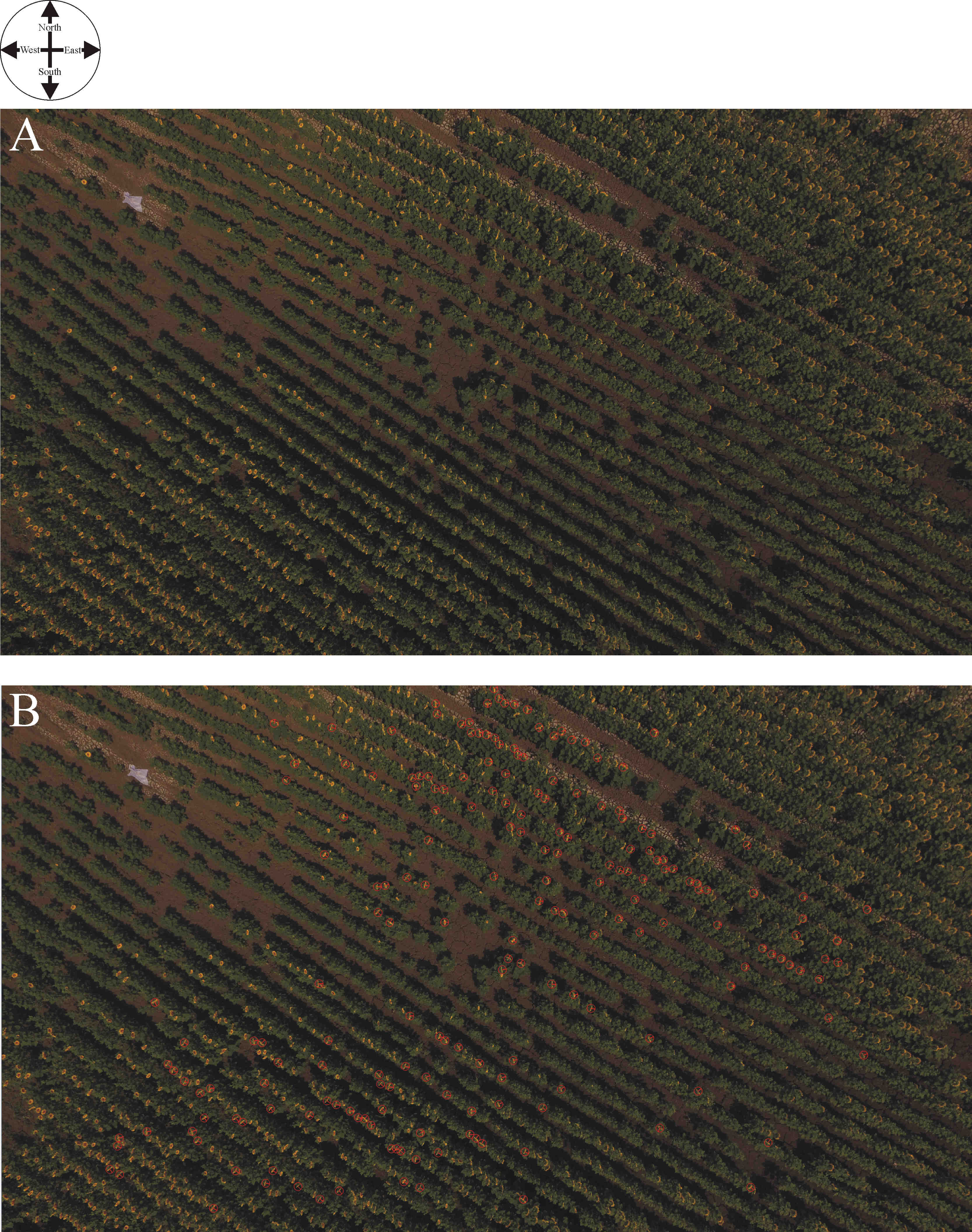


**Supplementary Figure S5**: Drone photographs of the sunflower plantation at location 4 (Sződ 3, Supplementary Table S1) taken by Balázs Bernáth. (A) Original drone photo. (B) Drone photo in which the 200 evaluated sunflower heads are marked by red circles and the normal vectors of their inflorescences are depicted by a red bar.


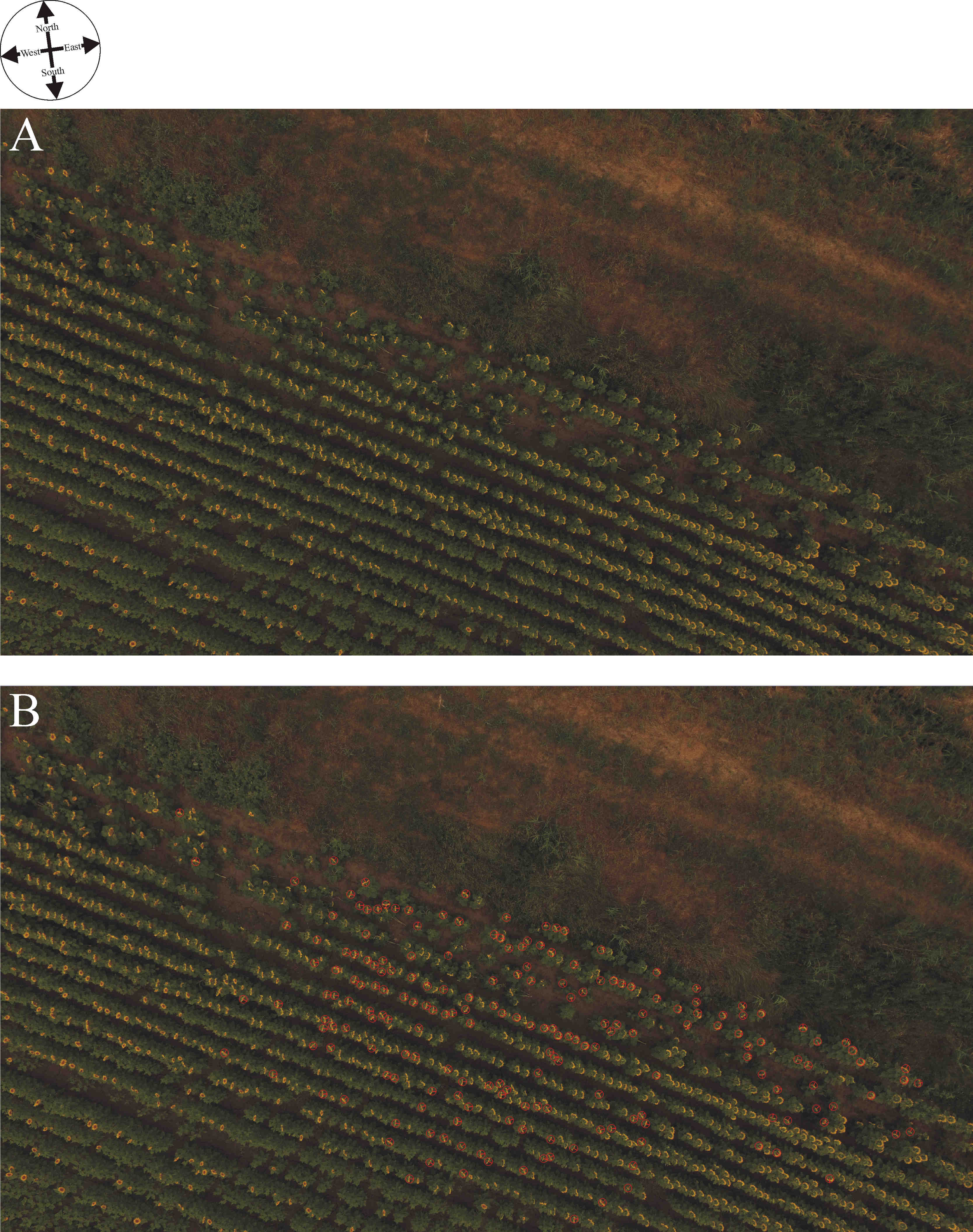


**Supplementary Figure S6**: Drone photographs of the sunflower plantation at location 5 (Sződ 4, Supplementary Table S1) taken by Balázs Bernáth. (A) Original drone photo. (B) Drone photo in which the 200 evaluated sunflower heads are marked by red circles and the normal vectors of their inflorescences are depicted by a red bar.


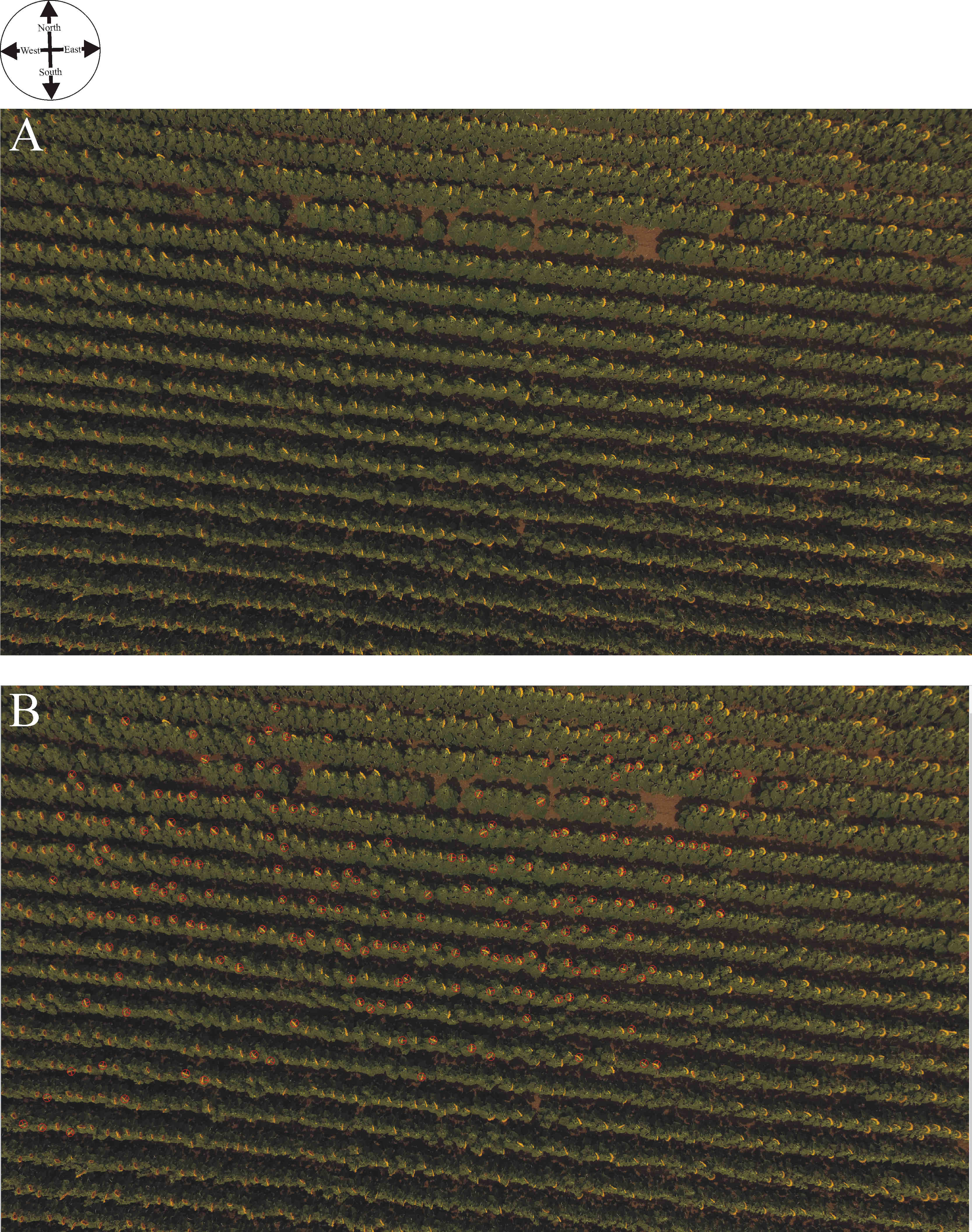


**Supplementary Figure S7**: Drone photographs of the sunflower plantation at location 6 (Vácduka 1, Supplementary Table S1) taken by Balázs Bernáth. (A) Original drone photo. (B) Drone photo in which the 200 evaluated sunflower heads are marked by red circles and the normal vectors of their inflorescences are depicted by a red bar.


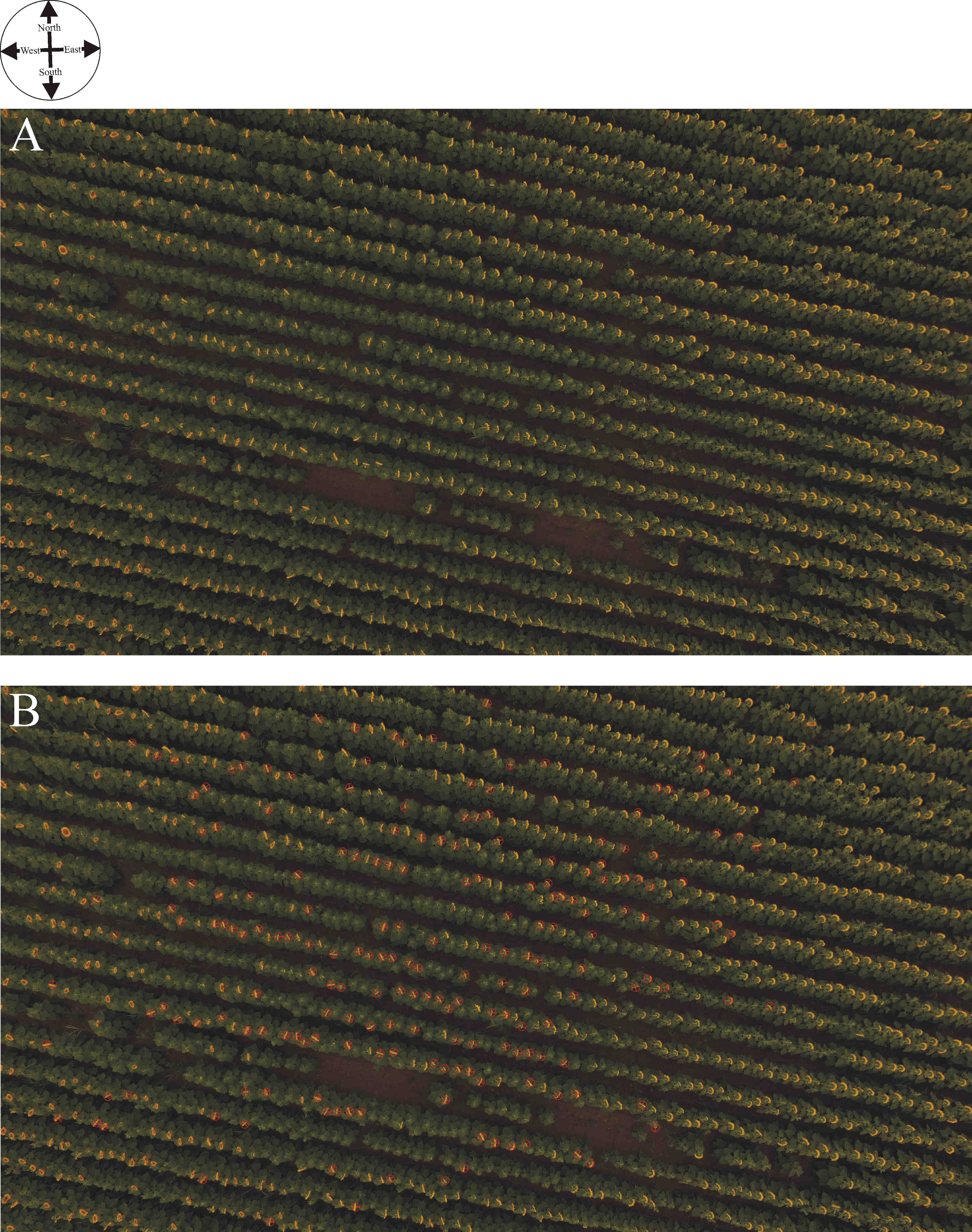


**Supplementary Figure S8**: Drone photographs of the sunflower plantation at location 7 (Vácduka 2, Supplementary Table S1) taken by Balázs Bernáth. (A) Original drone photo. (B) Drone photo in which the 200 evaluated sunflower heads are marked by red circles and the normal vectors of their inflorescences are depicted by a red bar.


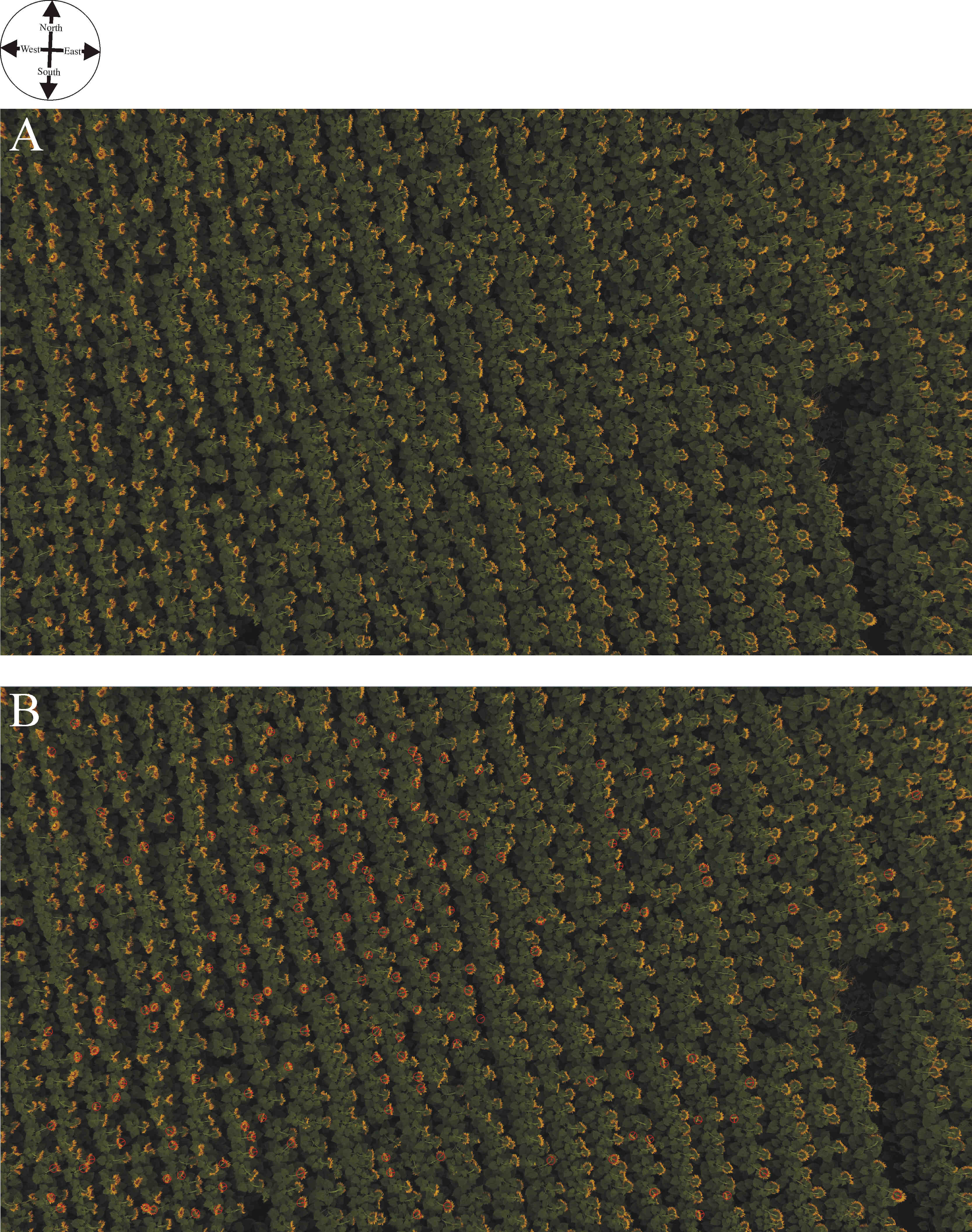


**Supplementary Figure S9**: Drone photographs of the sunflower plantation at location 8 (Környe 1, Supplementary Table S1) taken by Balázs Bernáth. (A) Original drone photo. (B) Drone photo in which the 200 evaluated sunflower heads are marked by red circles and the normal vectors of their inflorescences are depicted by a red bar.


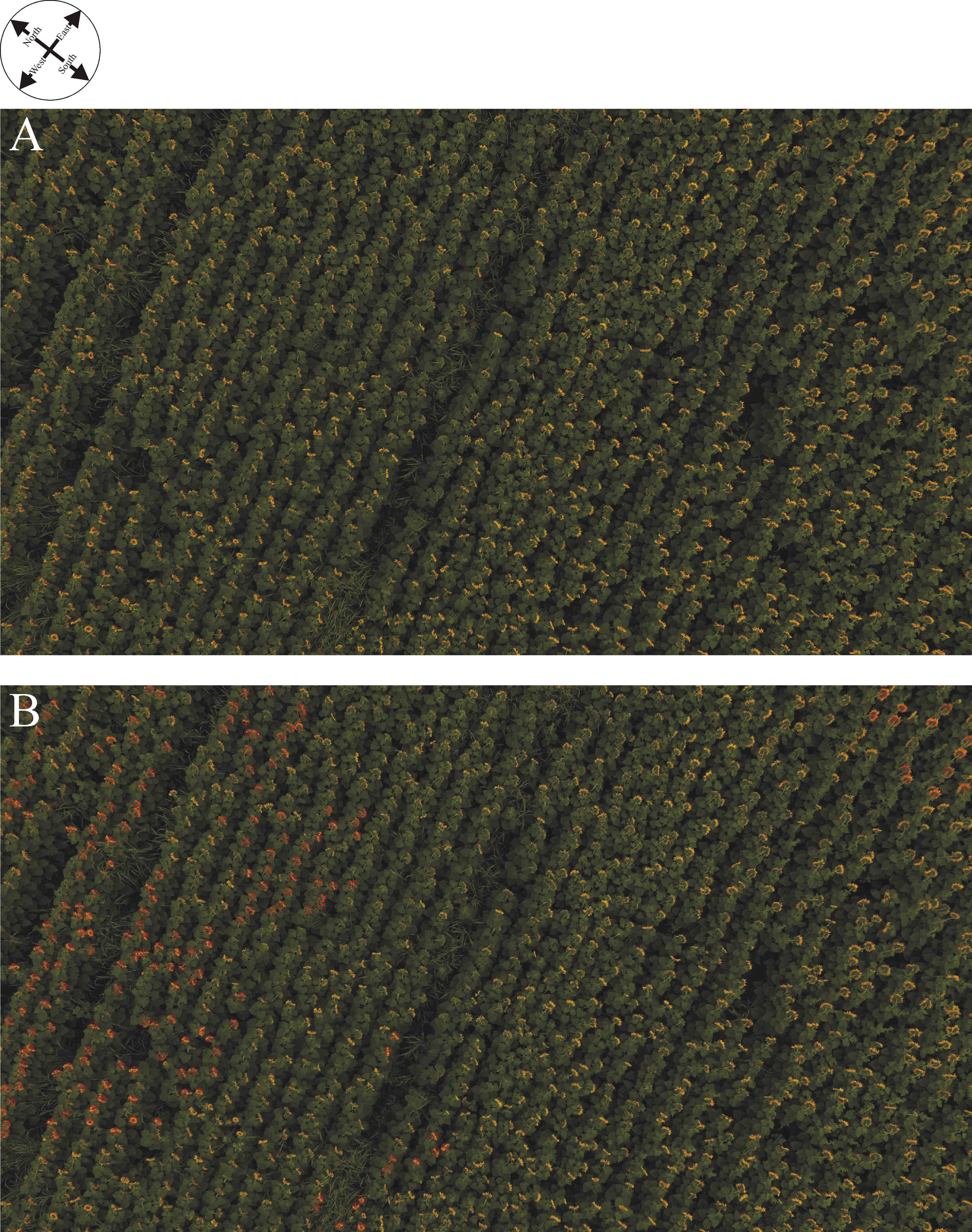


**Supplementary Figure S10**: Drone photographs of the sunflower plantation at location 9 (Környe 2, Supplementary Table S1) taken by Balázs Bernáth. (A) Original drone photo. (B) Drone photo in which the 200 evaluated sunflower heads are marked by red circles and the normal vectors of their inflorescences are depicted by a red bar.


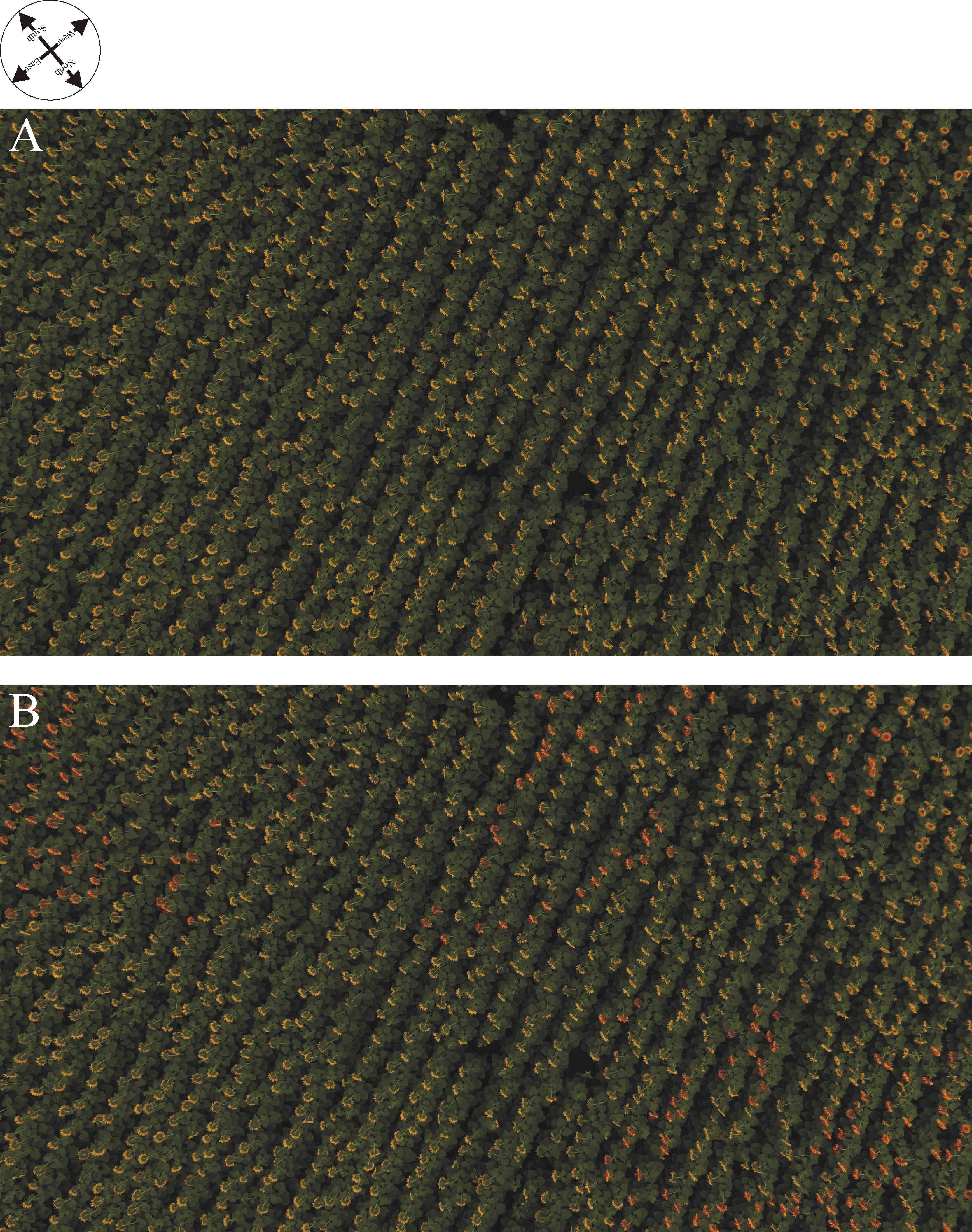


**Supplementary Figure S11**: Drone photographs of the sunflower plantation at location 10 (Környe 3, Supplementary Table S1) taken by Balázs Bernáth. (A) Original drone photo. (B) Drone photo in which the 200 evaluated sunflower heads are marked by red circles and the normal vectors of their inflorescences are depicted by a red bar.


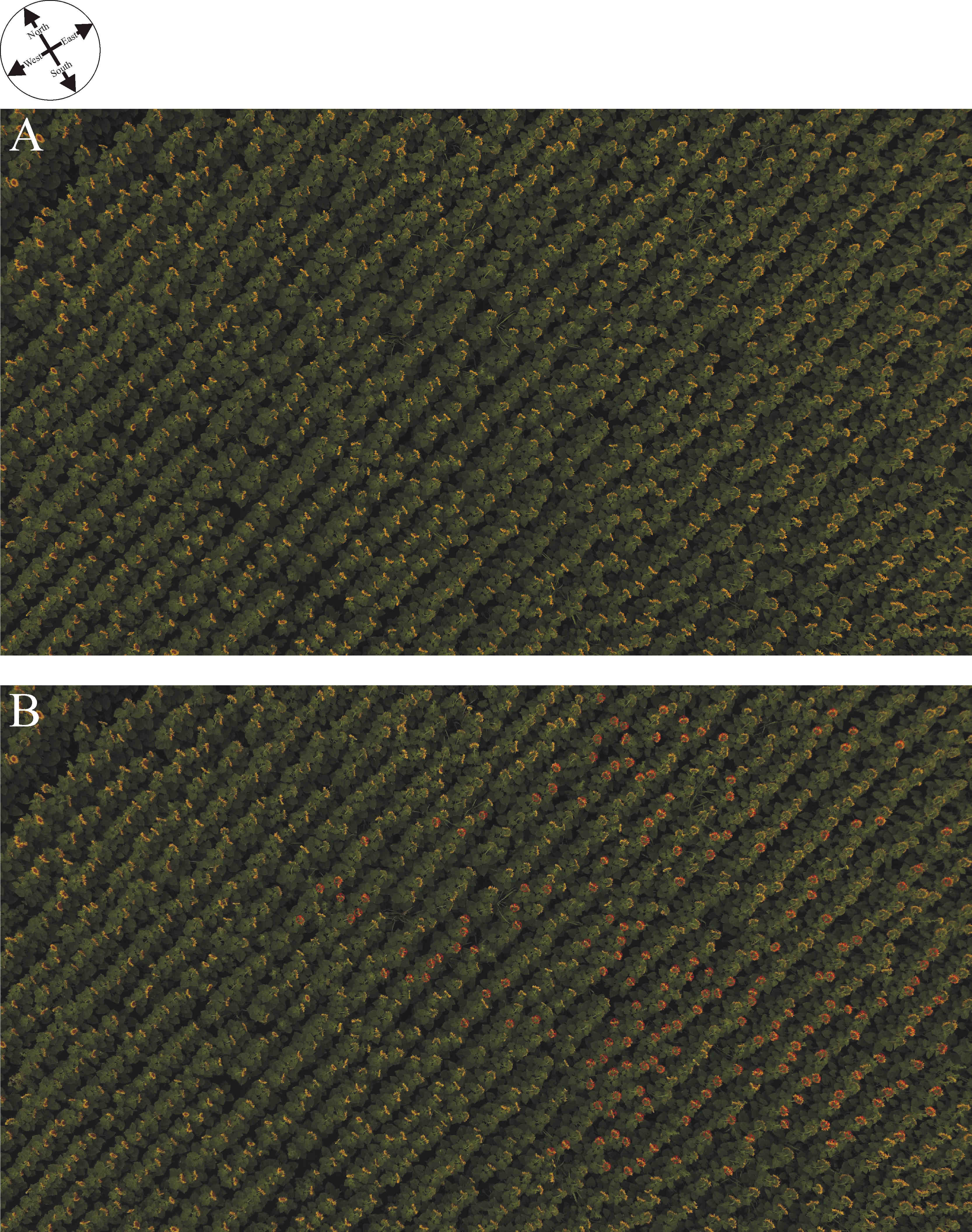


**Supplementary Figure S12**: Drone photographs of the sunflower plantation at location 11 (Környe 4, Supplementary Table S1) taken by Balázs Bernáth. (A) Original drone photo. (B) Drone photo in which the 200 evaluated sunflower heads are marked by red circles and the normal vectors of their inflorescences are depicted by a red bar.


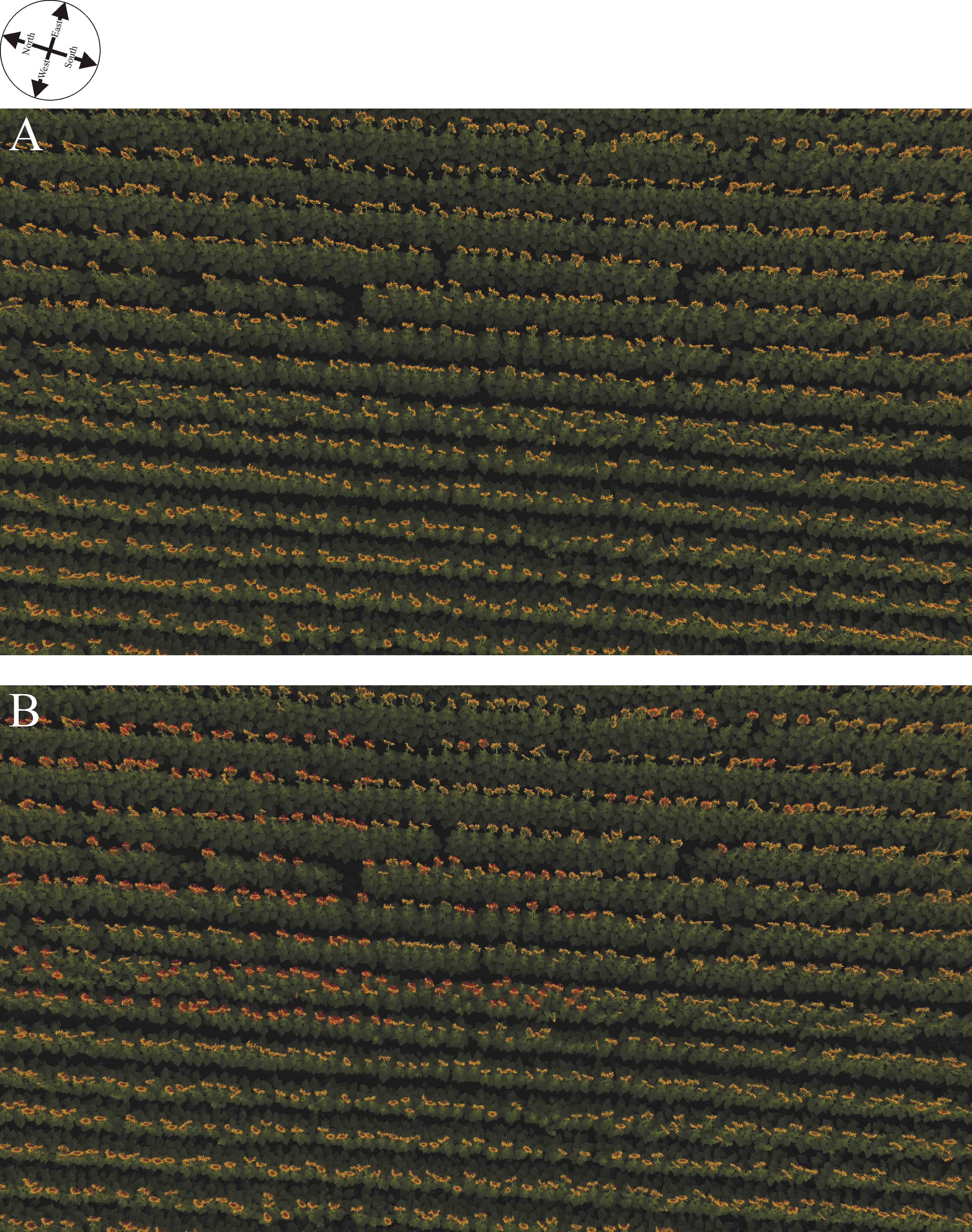


**Supplementary Figure S13**: Drone photographs of the sunflower plantation at location 12 (Környe 5, Supplementary Table S1) taken by Balázs Bernáth. (A) Original drone photo. (B) Drone photo in which the 200 evaluated sunflower heads are marked by red circles and the normal vectors of their inflorescences are depicted by a red bar.


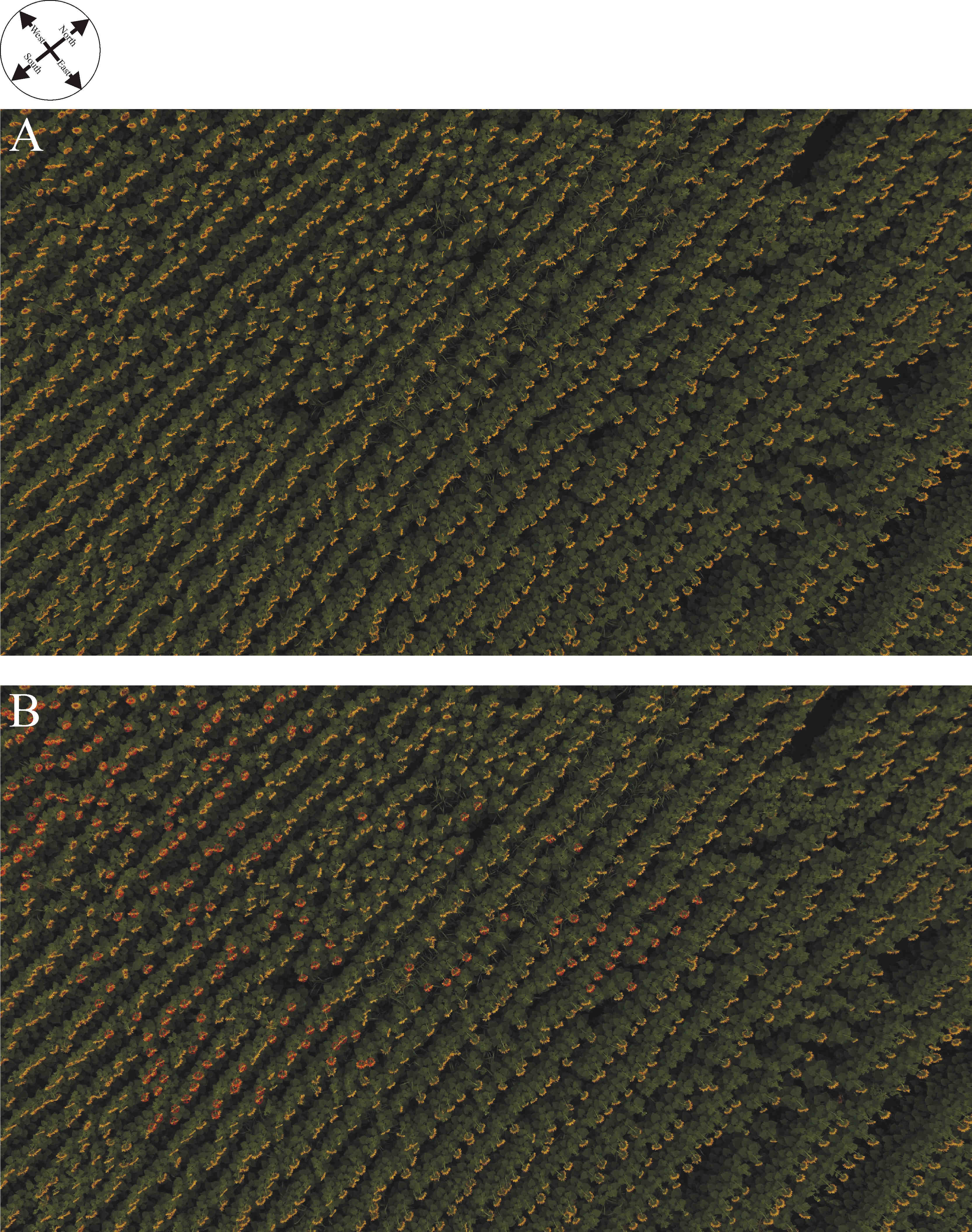


**Supplementary Figure S14**: Drone photographs of the sunflower plantation at location 13 (Környe 6, Supplementary Table S1) taken by Balázs Bernáth. (A) Original drone photo. (B) Drone photo in which the 200 evaluated sunflower heads are marked by red circles and the normal vectors of their inflorescences are depicted by a red bar.


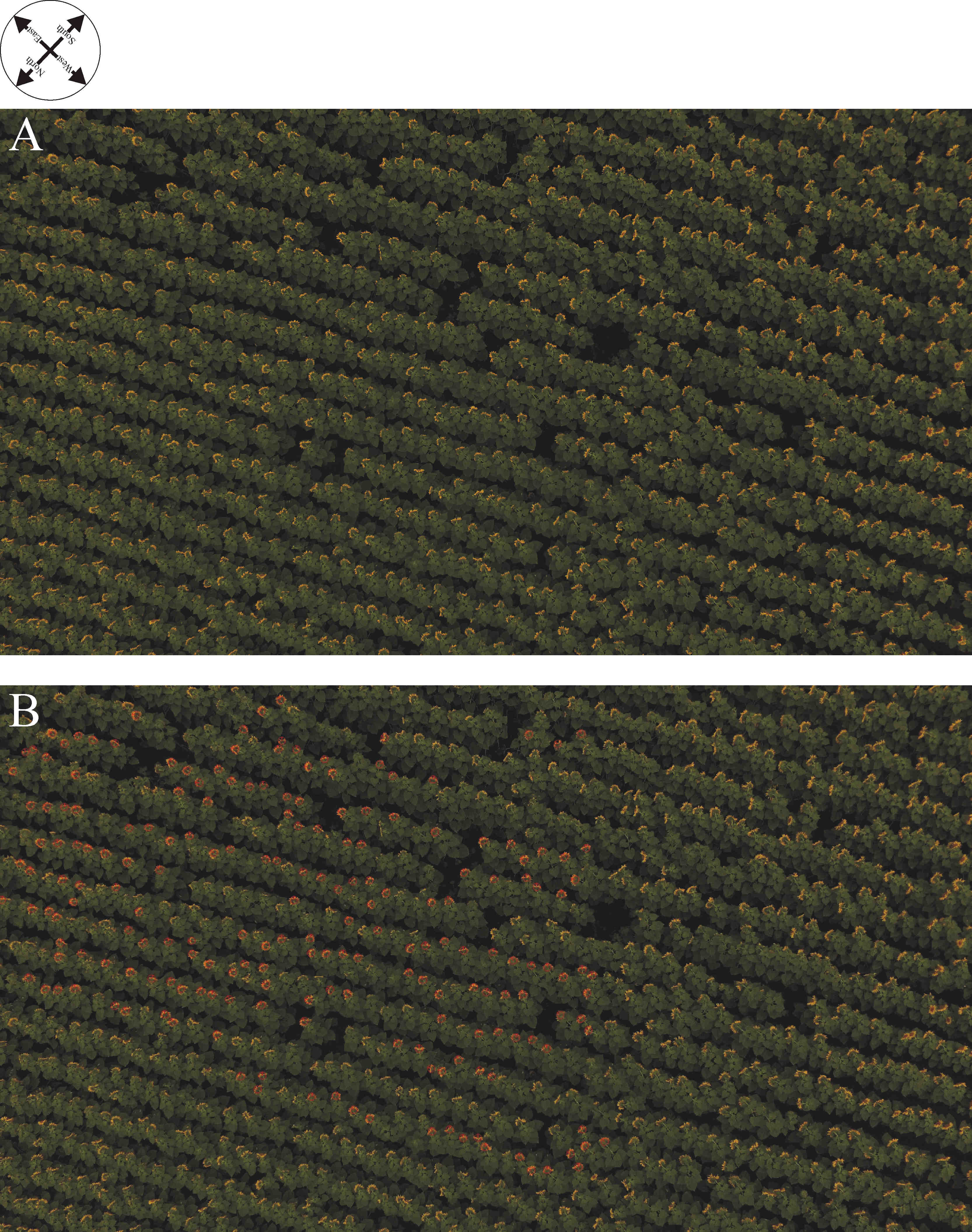


**Supplementary Figure S15**: Drone photographs of the sunflower plantation at location 14 (Környe 7, Supplementary Table S1) taken by Balázs Bernáth. (A) Original drone photo. (B) Drone photo in which the 200 evaluated sunflower heads are marked by red circles and the normal vectors of their inflorescences are depicted by a red bar.


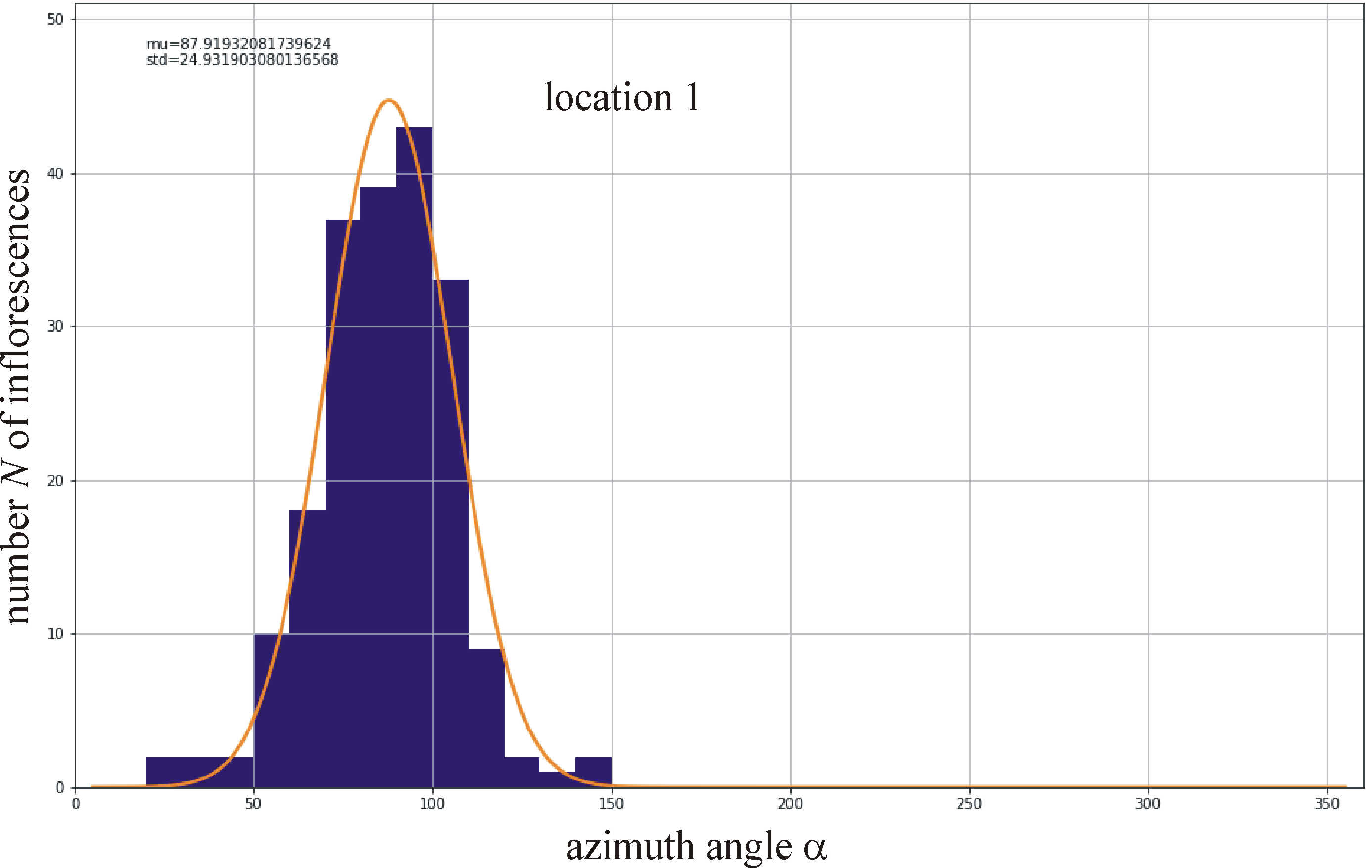


**Supplementary Figure S16:** Distribution of the azimuth angle α (measured clockwise from north) of the normal vector of randomly selected 200 mature sunflower inflorescences at location 1 (Kiskunhalas, Supplementary Table S1) determined in the drone photograph of Supplementary Fig. S1, where *N* is the number of inflorescences. The Gaussian curve (characterized by peak azimuth α_average_ = 87.9^o^ and standard deviation Δα_SD_ = ±24.9^o^) is fitted to the *N*(α) graph. α_sunrise_ = 56.14^o^ is the azimuth of local sunrise.


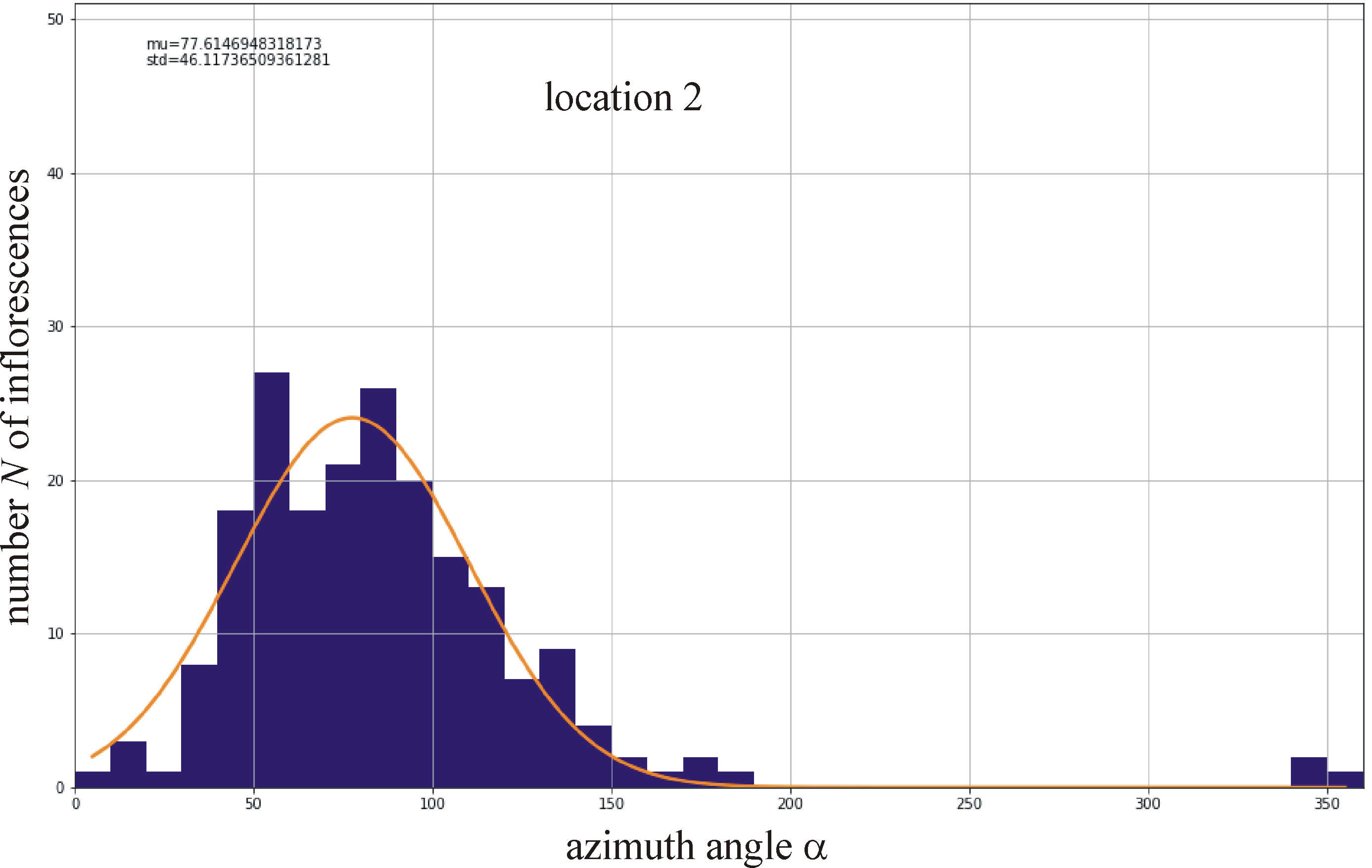


**Supplementary Figure S17:** Distribution of the azimuth angle α (measured clockwise from north) of the normal vector of randomly selected 200 mature sunflower inflorescences at location 2 (Sződ 1, Supplementary Table S1) determined in the drone photograph of Supplementary Fig. S2. The Gaussian curve (characterized by the peak azimuth α_average_ = 77.6^o^ and standard deviation Δα_SD_ = ±46.1^o^) is fitted to the *N*(α) graph. α_sunrise_ = 56.66^o^ is the azimuth of local sunrise.


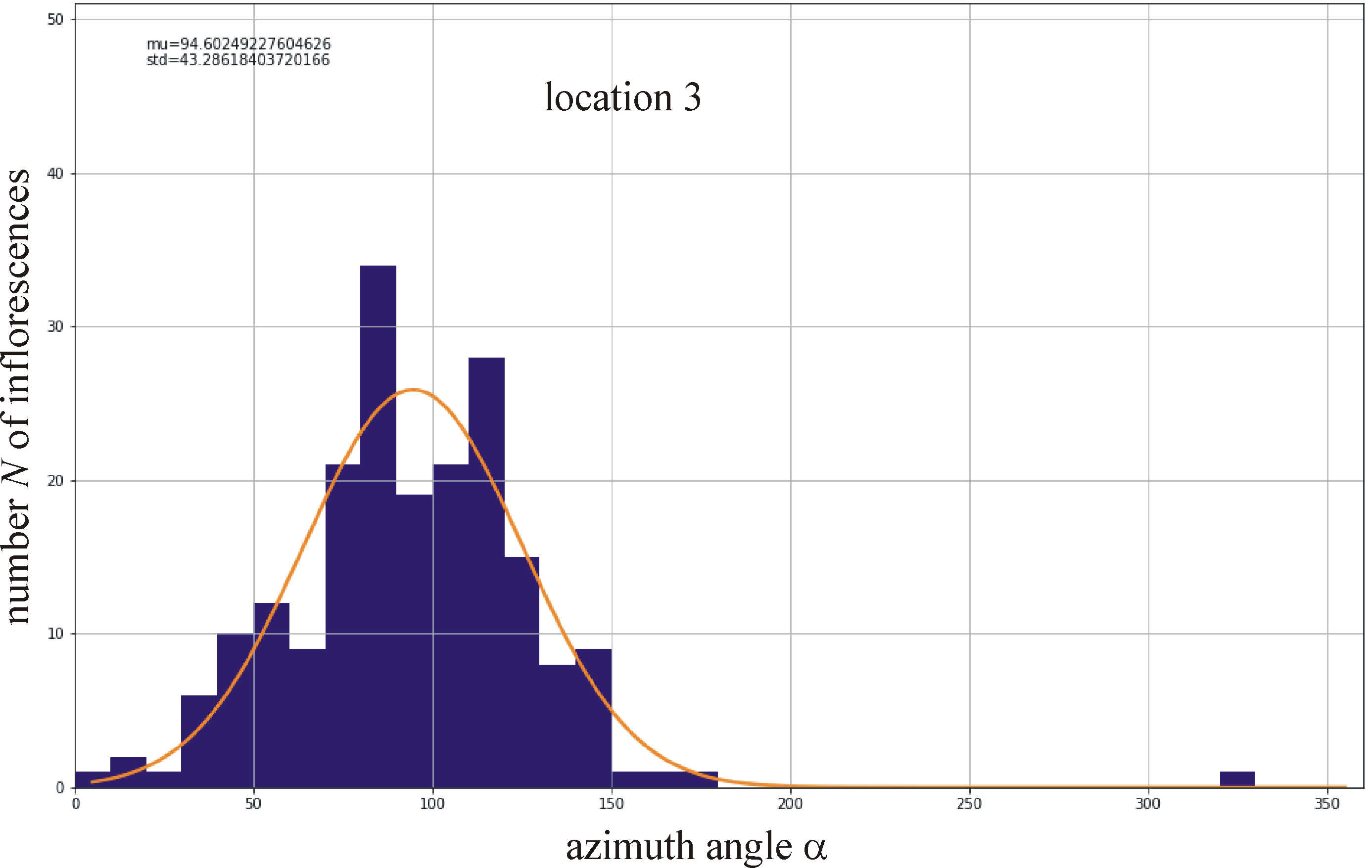


**Supplementary Figure S18:** Distribution of the azimuth angle α (measured clockwise from north) of the normal vector of randomly selected 200 mature sunflower inflorescences at location 3 (Sződ 2, Supplementary Table S1) determined in the drone photograph of Supplementary Fig. S3. The Gaussian curve (characterized by peak azimuth α_average_ = 94.6^o^ and standard deviation Δα_SD_ = ±43.3^o^) is fitted to the *N*(α) graph. α_sunrise_ = 56.66^o^ is the azimuth of local sunrise.


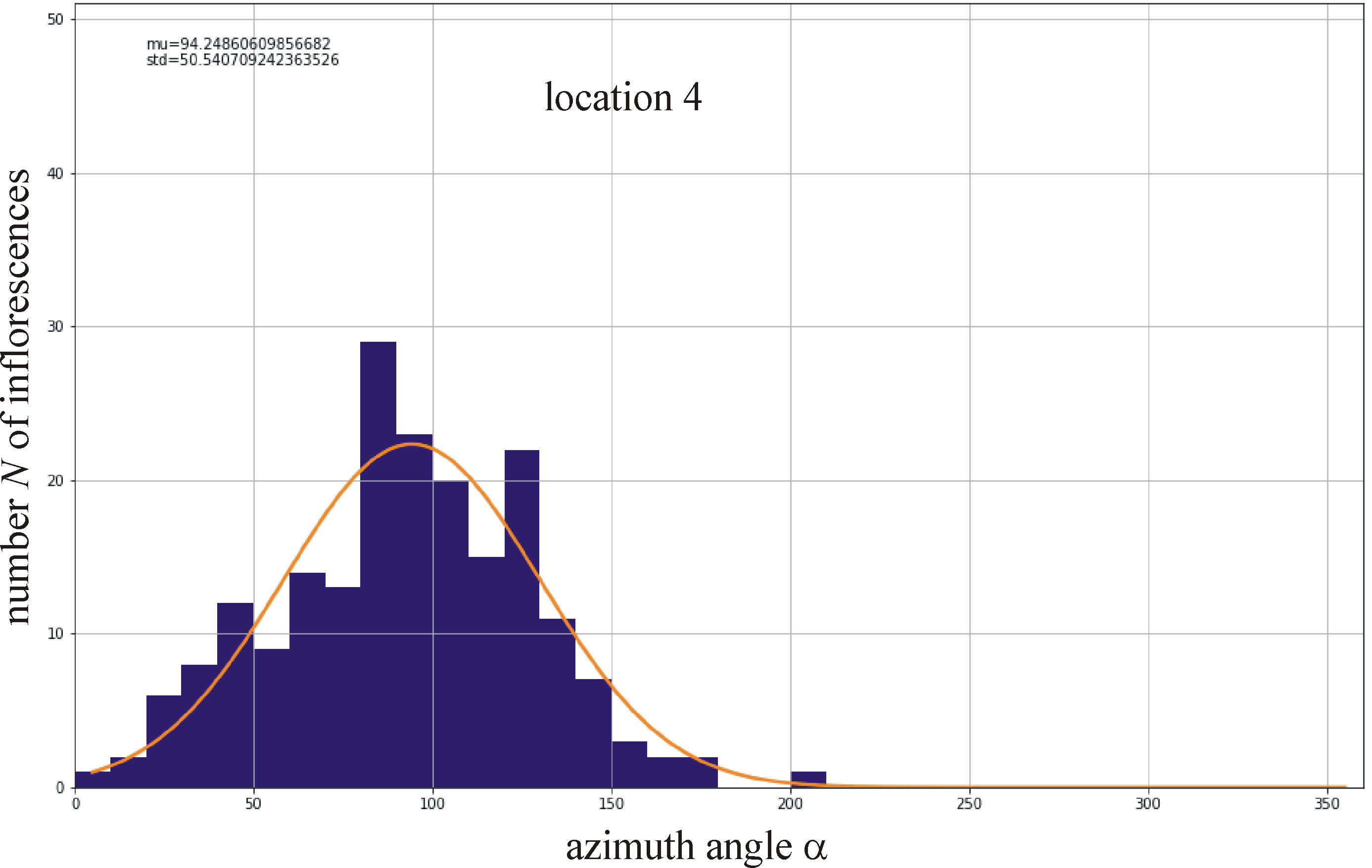


**Supplementary Figure S19:** Distribution of the azimuth angle α (measured clockwise from north) of the normal vector of randomly selected 200 mature sunflower inflorescences at location 4 (Sződ 3, Supplementary Table S1) determined in the drone photograph of Supplementary Fig. S4. The Gaussian curve (characterized by peak azimuth α_average_ = 94.2^o^ and standard deviation Δα_SD_ = ±50.5^o^) is fitted to the *N*(α) graph. α_sunrise_ = 56.66^o^ is the azimuth of local sunrise.


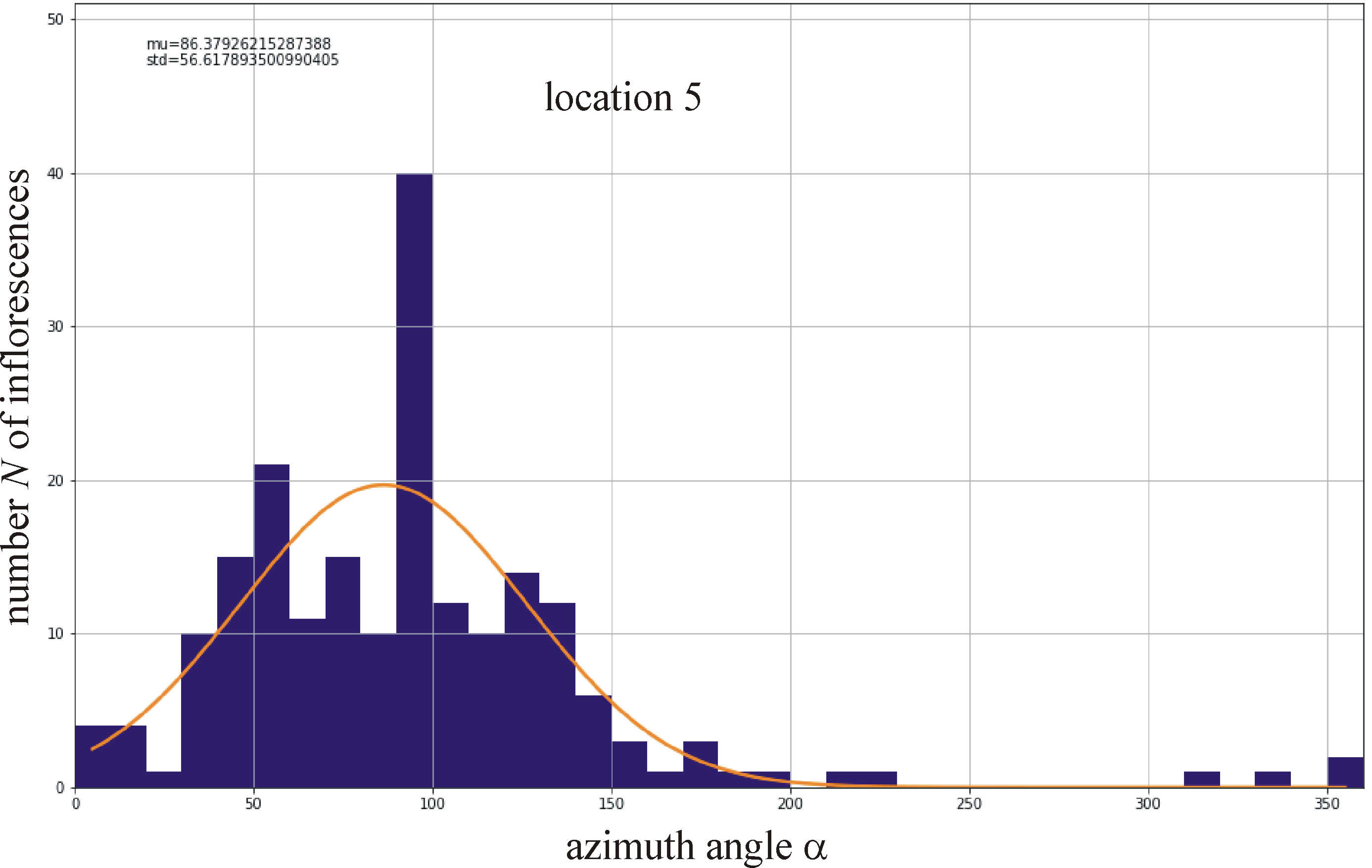


**Supplementary Figure S20:** Distribution of the azimuth angle α (measured clockwise from north) of the normal vector of randomly selected 200 mature sunflower inflorescences at location 5 (Sződ 4, Supplementary Table S1) determined in the drone photograph of Supplementary Fig. S5. The Gaussian curve (characterized by peak azimuth α_average_ = 86.4^o^ and standard deviation Δα_SD_ = ±56.6^o^) is fitted to the *N*(α) graph. α_sunrise_ = 56.66^o^ is the azimuth of local sunrise.


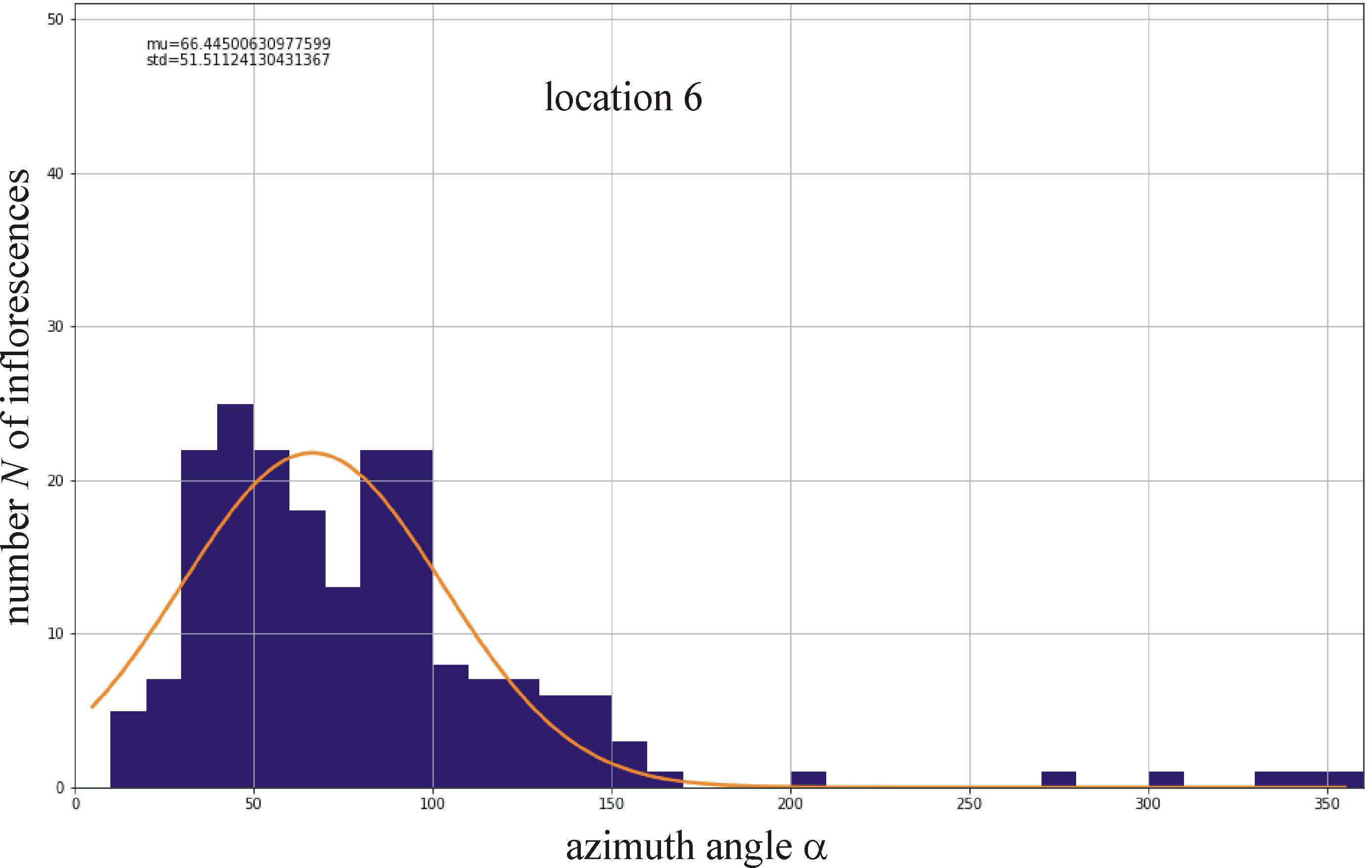


**Supplementary Figure S21:** Distribution of the azimuth angle α (measured clockwise from north) of the normal vector of randomly selected 200 mature sunflower inflorescences at location 6 (Vácduka 1, Supplementary Table S1) determined in the drone photograph of Supplementary Fig. S6. The Gaussian curve (characterized by peak azimuth α_average_ = 66.4^o^ and standard deviation Δα_SD_ = ±51.5^o^) is fitted to the *N*(α) graph. α_sunrise_ = 56.65^o^ is the azimuth of local sunrise.


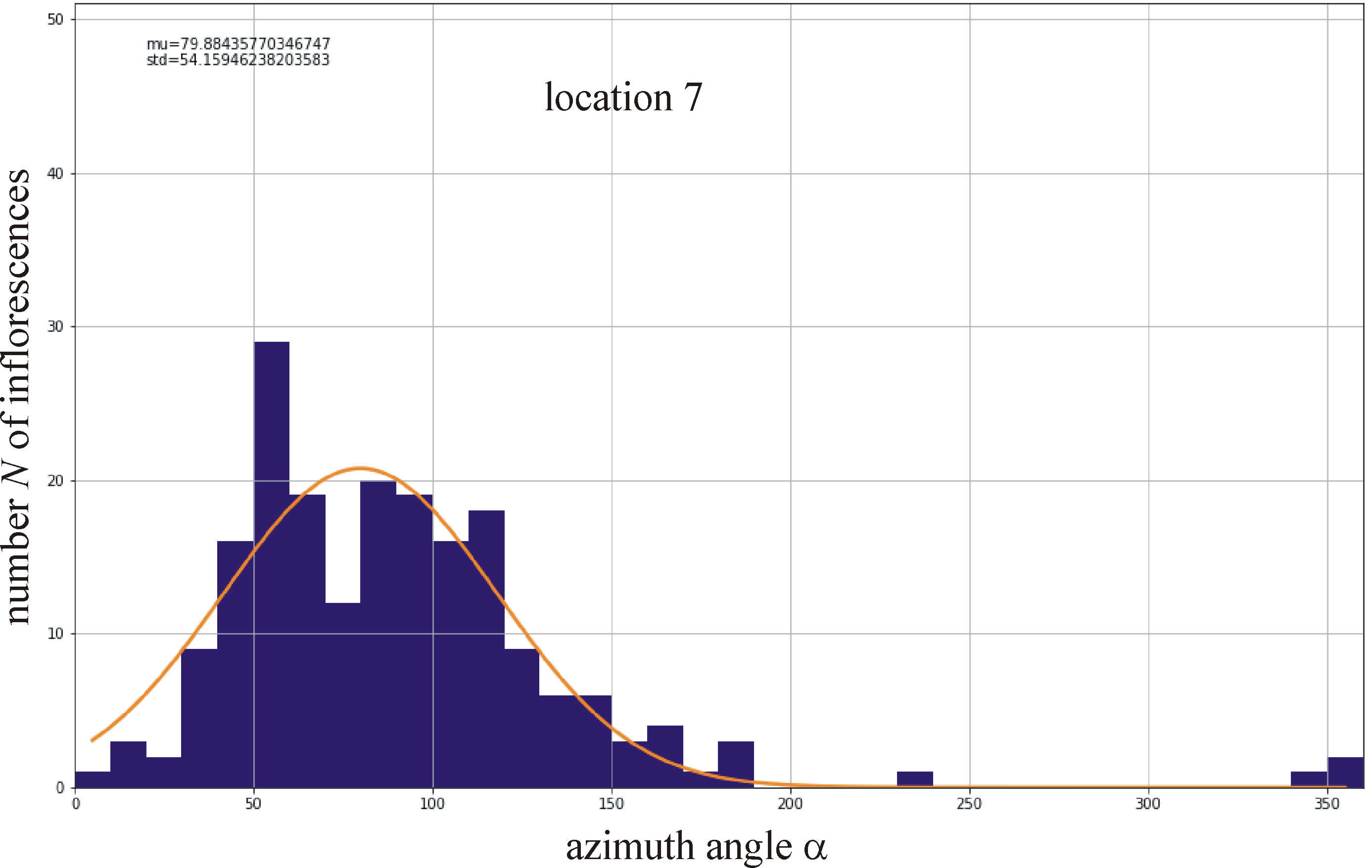


**Supplementary Figure S22:** Distribution of the azimuth angle α (measured clockwise from north) of the normal vector of randomly selected 200 mature sunflower inflorescences at location 7 (Vácduka 2, Supplementary Table S1) determined in the drone photograph of Supplementary Fig. S7. The Gaussian curve (characterized by peak azimuth α_average_ = 79.9^o^ and standard deviation Δα_SD_ = ±54.2^o^) is fitted to the *N*(α) graph. α_sunrise_ = 56.65^o^ is the azimuth of local sunrise.


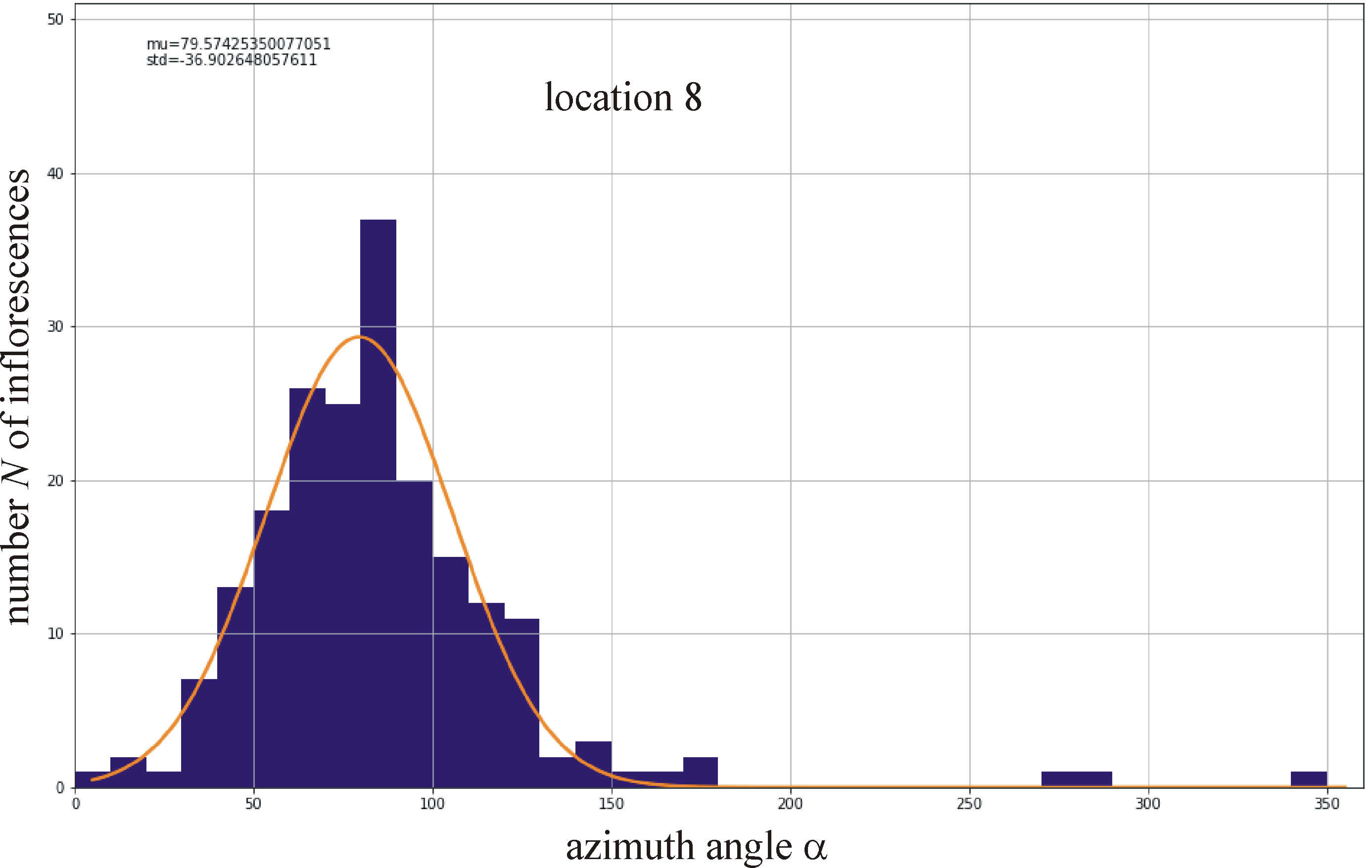


**Supplementary Figure S23:** Distribution of the azimuth angle α (measured clockwise from north) of the normal vector of randomly selected 200 mature sunflower inflorescences at location 8 (Környe 1, Supplementary Table S1) determined in the drone photograph of Supplementary Fig. S8. The Gaussian curve (characterized by peak azimuth α_average_ = 79.6^o^ and standard deviation Δα_SD_ = ±36.9^o^) is fitted to the *N*(α) graph. α_sunrise_ = 57.52^o^ is the azimuth of local sunrise.


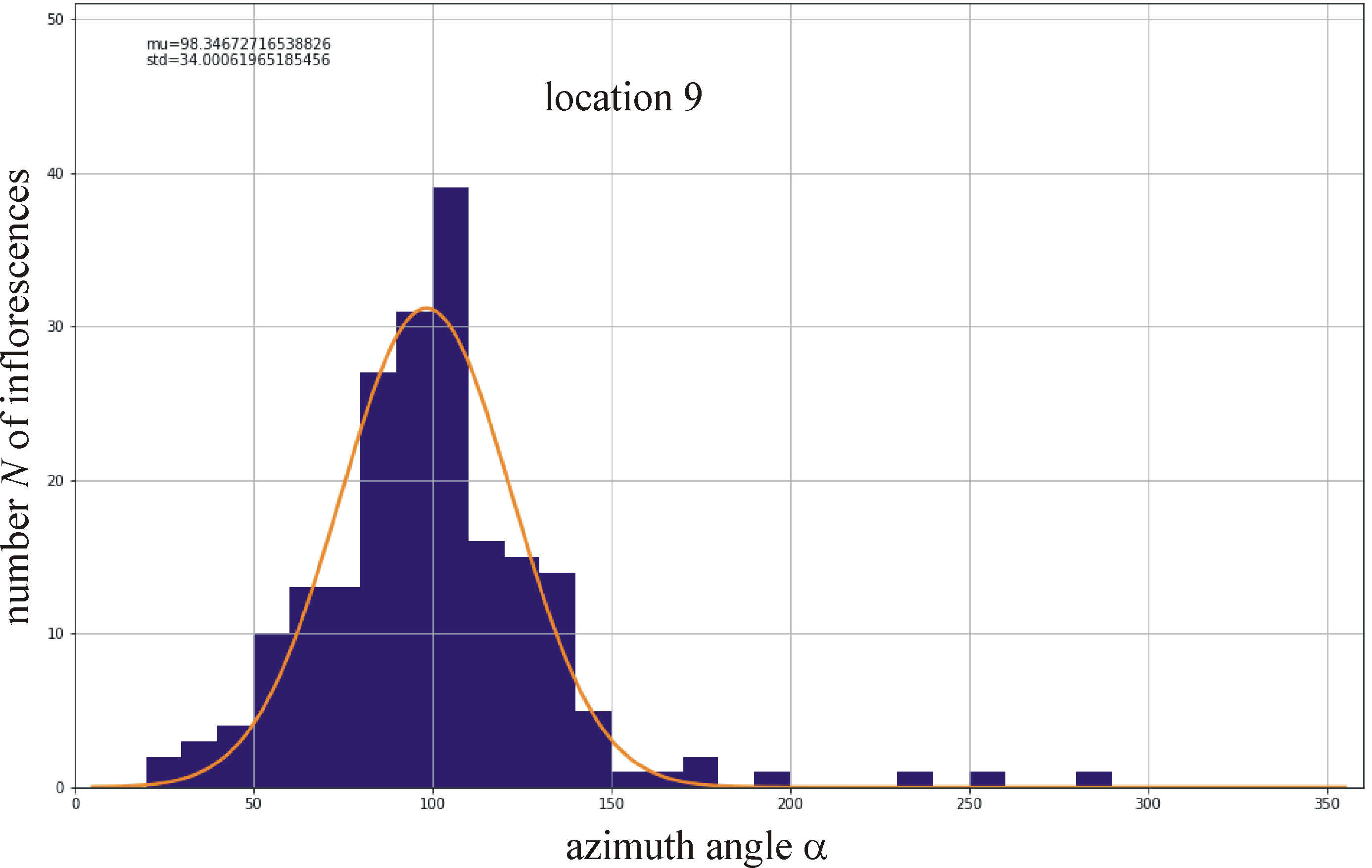


**Supplementary Figure S24:** Distribution of the azimuth angle α (measured clockwise from north) of the normal vector of randomly selected 200 mature sunflower inflorescences at location 9 (Környe 2, Supplementary Table S1) determined in the drone photograph of Supplementary Fig. S9. The Gaussian curve (characterized by peak azimuth α_average_ = 98.3^o^ and standard deviation Δα_SD_ = ±34.0^o^) is fitted to the *N*(α) graph. α_sunrise_ = 57.52^o^ is the azimuth of local sunrise.


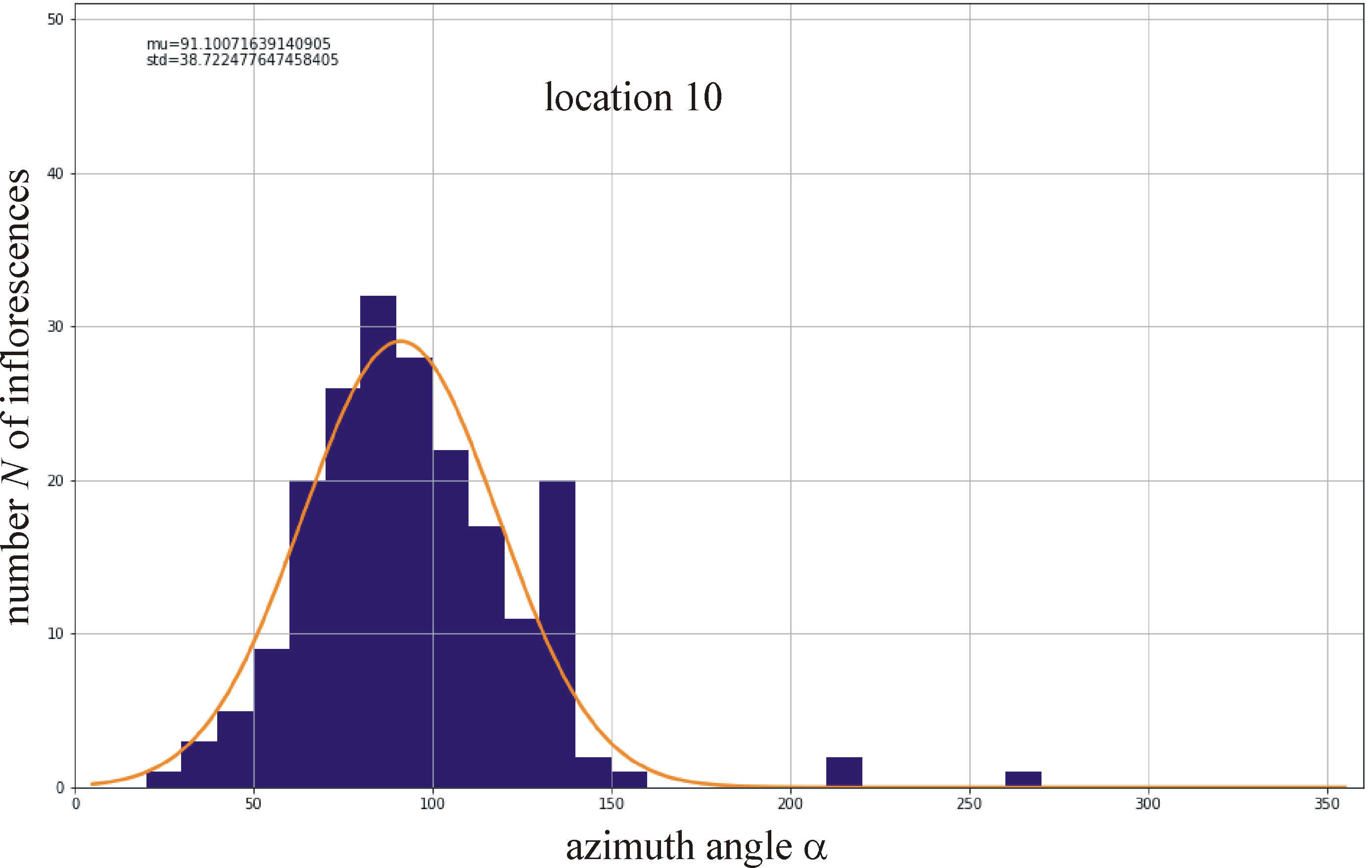


**Supplementary Figure S25:** Distribution of the azimuth angle α (measured clockwise from north) of the normal vector of randomly selected 200 mature sunflower inflorescences at location 10 (Környe 3, Supplementary Table S1) determined in the drone photograph of Supplementary Fig. S10. The Gaussian curve (characterized by peak azimuth α_average_ = 91.1^o^ and standard deviation Δα_SD_ = ±38.7^o^) is fitted to the *N*(α) graph. α_sunrise_ = 57.52^o^ is the azimuth of local sunrise.


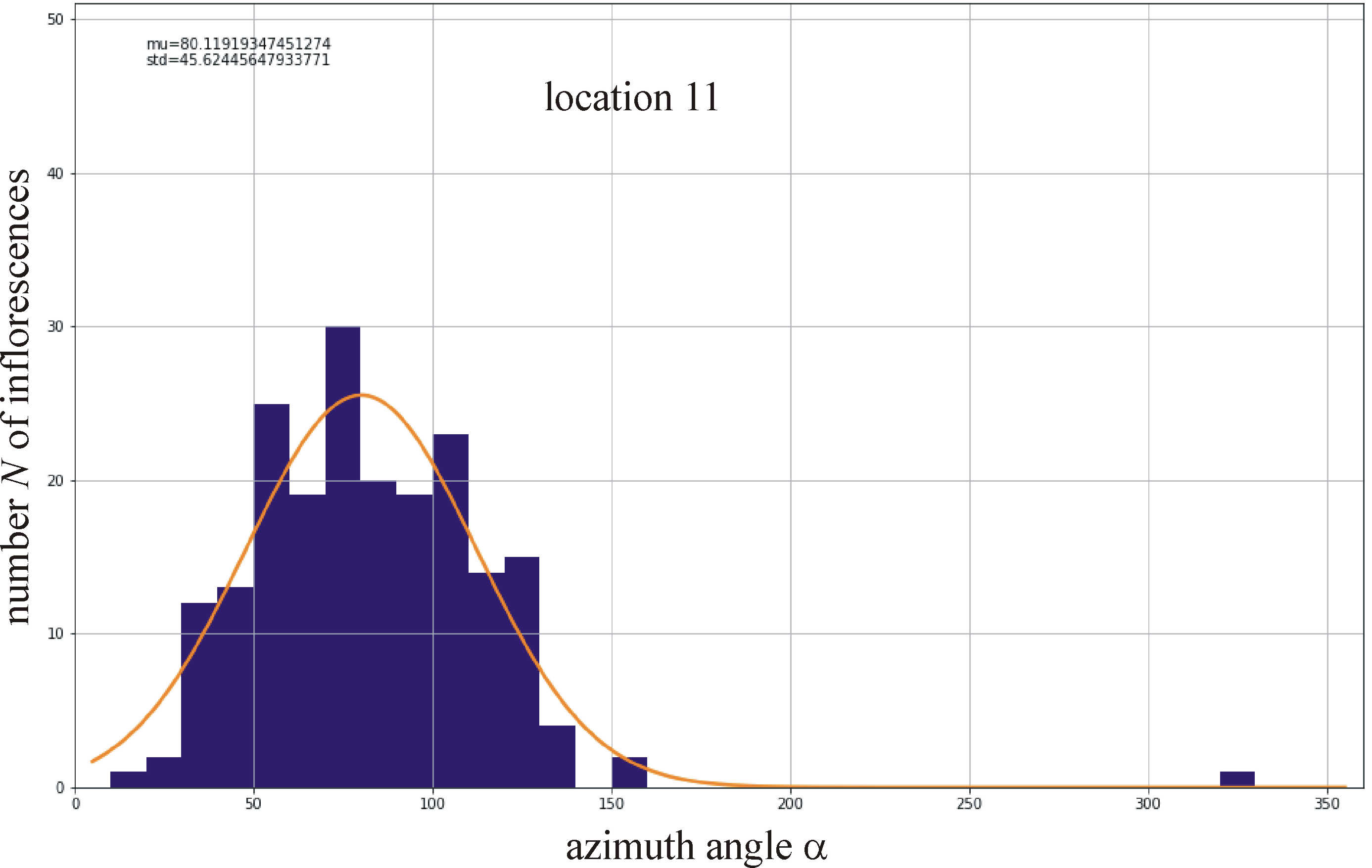


**Supplementary Figure S26:** Distribution of the azimuth angle α (measured clockwise from north) of the normal vector of randomly selected 200 mature sunflower inflorescences at location 11 (Környe 4, Supplementary Table S1) determined in the drone photograph of Supplementary Fig. S11. The Gaussian curve (characterized by peak azimuth α_average_ = 80.1^o^ and standard deviation Δα_SD_ = ±45.6^o^) is fitted to the *N*(α) graph. α_sunrise_ = 57.52^o^ is the azimuth of local sunrise.


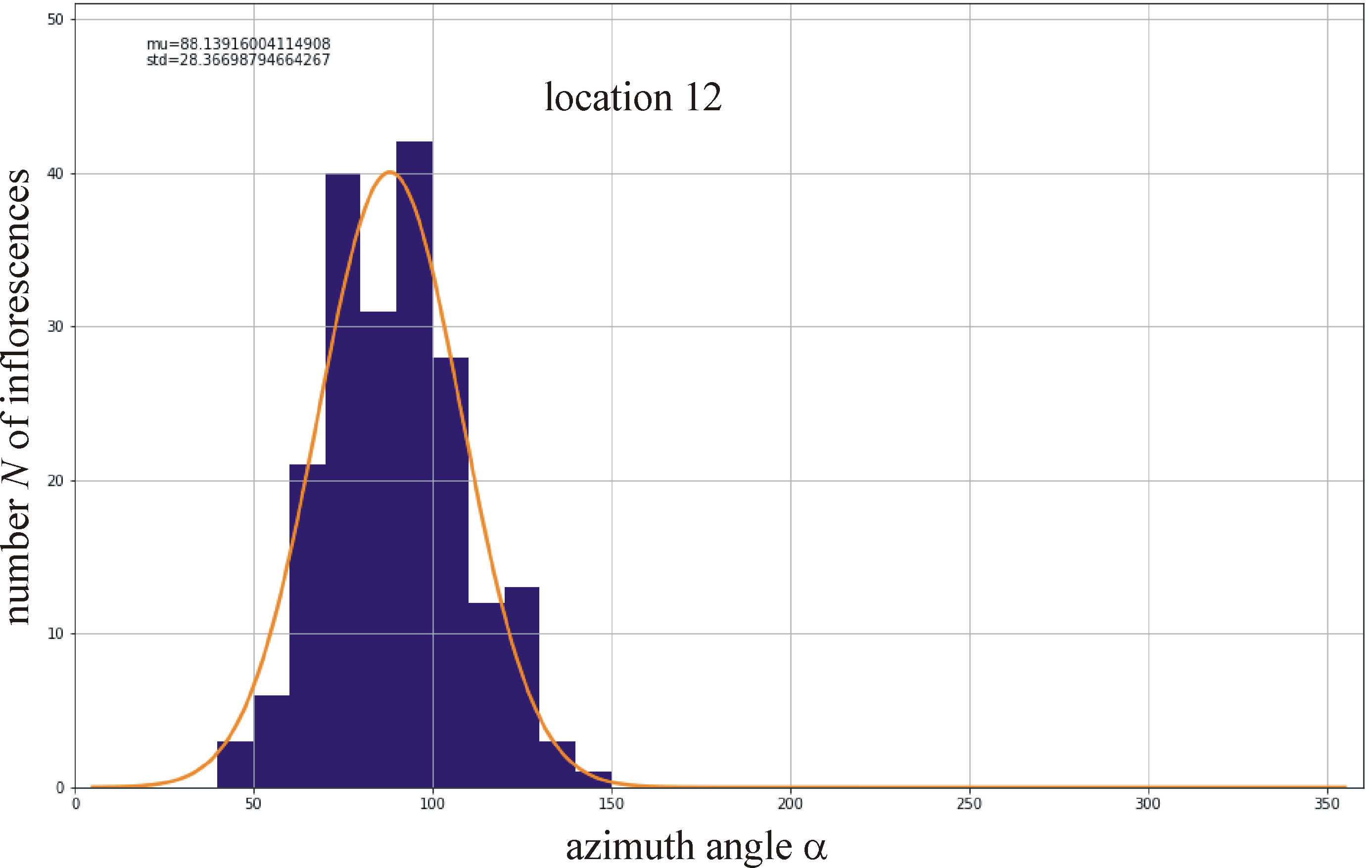


**Supplementary Figure S27:** Distribution of the azimuth angle α (measured clockwise from north) of the normal vector of randomly selected 200 mature sunflower inflorescences at location 12 (Környe 5, Supplementary Table S1) determined in the drone photograph of Supplementary Fig. S12. The Gaussian curve (characterized by peak azimuth α_average_ = 88.1^o^ and standard deviation Δα_SD_ = ±28.4^o^) is fitted to the *N*(α) graph. α_sunrise_ = 57.52^o^ is the azimuth of local sunrise.


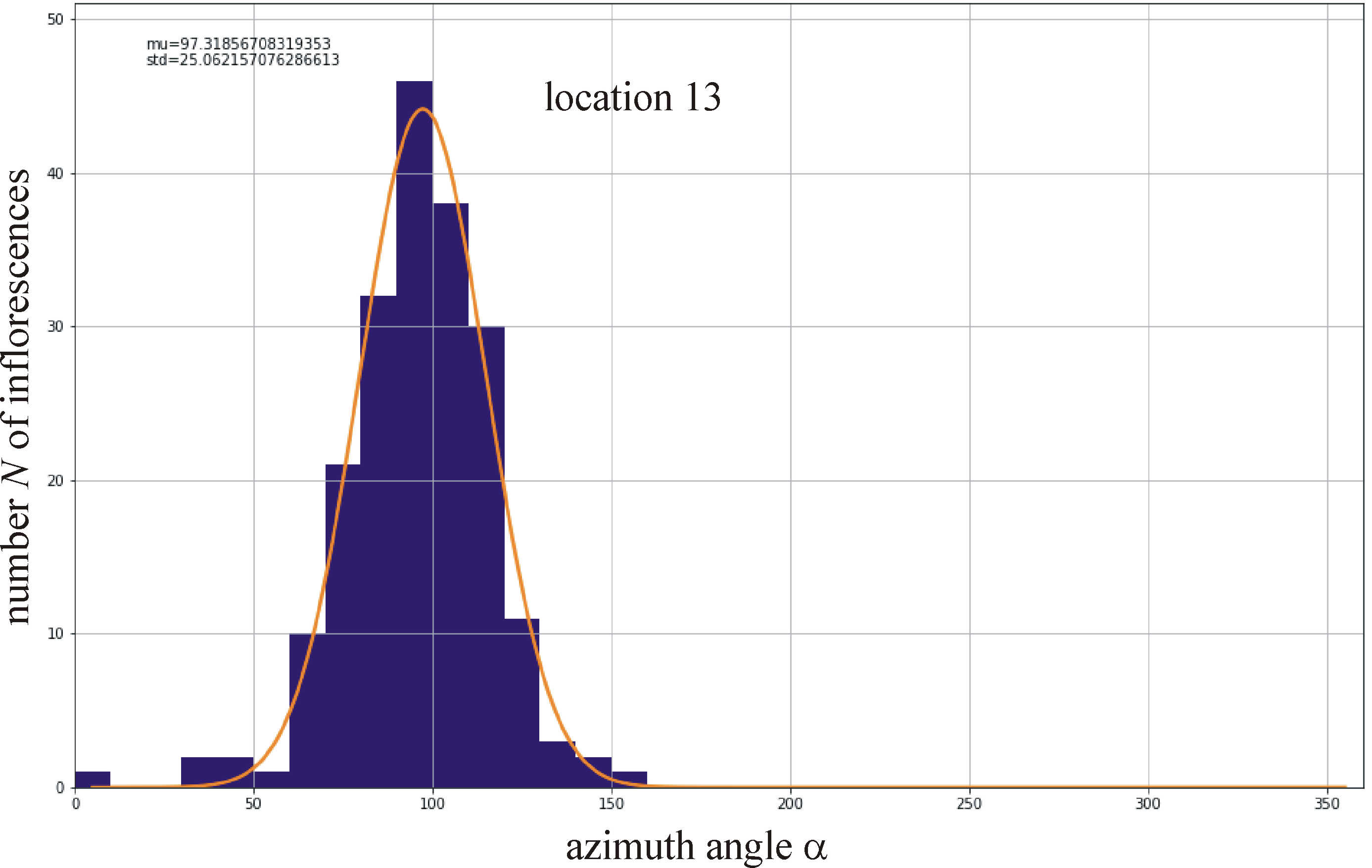


**Supplementary Figure S28:** Distribution of the azimuth angle α (measured clockwise from north) of the normal vector of randomly selected 200 mature sunflower inflorescences at location 13 (Környe 6, Supplementary Table S1) determined in the drone photograph of Supplementary Fig. S13. The Gaussian curve (characterized by peak azimuth α_average_ = 97.3^o^ and standard deviation Δα_SD_ = ±25.1^o^) is fitted to the *N*(α) graph. α_sunrise_ = 57.52^o^ is the azimuth of local sunrise.


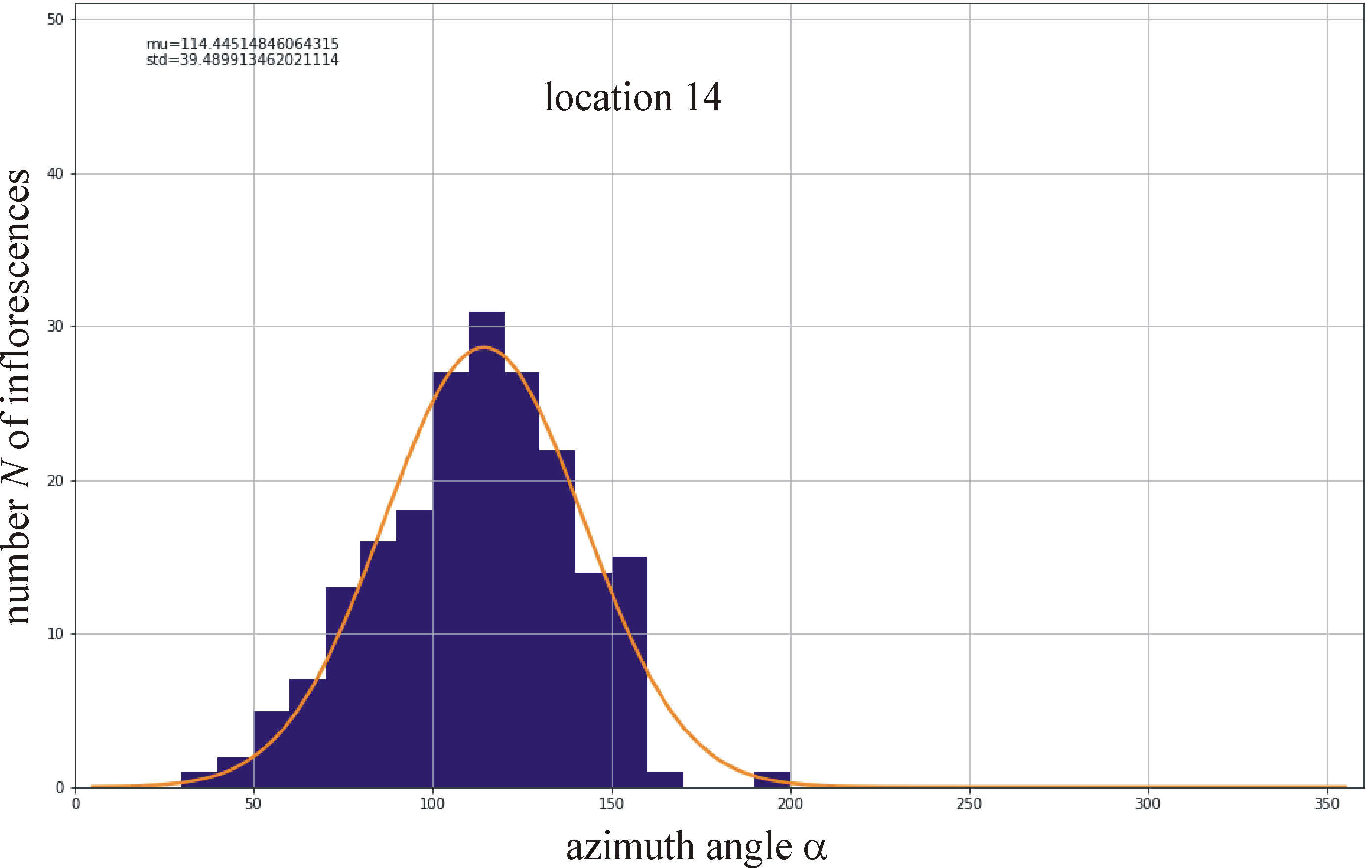


**Supplementary Figure S29:** Distribution of the azimuth angle α (measured clockwise from north) of the normal vector of randomly selected 200 mature sunflower inflorescences at location 14 (Környe 7, Supplementary Table S1) determined in the drone photograph of Supplementary Fig. S14. The Gaussian curve (characterized by peak azimuth α_average_ = 114.4^o^ and standard deviation Δα_SD_ = ±39.5^o^) is fitted to the *N*(α) graph. α_sunrise_ = 57.55^o^ is the azimuth of local sunrise.
